# Equal Provision, Unequal Benefit: Social Class and the Conversion of Employer-Provided Digital Training into Worker Wellbeing Across 27 European Countries

**DOI:** 10.3390/bs16101899

**Published:** 2026-10-14

**Authors:** Asad Ul Islam Khan, Muhittin Kaplan, Volkan Öngel, Umaal Ahmed Khan

**Affiliations:** 1 Department of Economics, School of Business, Ibn Haldun University, Başakşehir, Istanbul 34494, Türkiye; muhittin.kaplan@ihu.edu.tr; 2 Department of Trade and Finance, Faculty of Economics and Administrative Sciences, Istanbul Beykent University, Istanbul 34475, Türkiye; volkanongel@beykent.edu.tr; 3 Department of Management, School of Business, Ibn Haldun University, Istanbul 34494, Türkiye; umaalkhan@ihu.edu.tr

**Keywords:** Artificial Intelligence (AI), employer-provided digital training, digital skills, worker wellbeing, social class, capability approach, job demands–resources (JD-R), structural equation modeling (SEM)

## Abstract

Employers increasingly provide training for working with digital technologies and artificial intelligence (AI), but it is unclear whether the same provision benefits all workers equally. Integrating the job demands–resources (JD-R) model with Sen’s capability approach, this study treats workplace AI exposure as a potential job demand, digital skills and employer-provided digital training as resources, capability-relevant wellbeing as the outcome, and social class as a proxy for the conversion conditions under which resources are used to ensure wellbeing. Structural equation models with ordinal indicators were estimated for 8065 employees in 27 EU countries (Special Eurobarometer 101.4), with country fixed effects, country-clustered and delete-one-country jackknife standard errors, and Random Forest and SHAP analyses as robustness checks. AI exposure was weakly and negatively associated with wellbeing (β = −0.073) and explained almost none of the variance in AI threat appraisal (R^2^ = 0.002). Employer-provided digital training (β = 0.209) and digital skills (β = 0.189) were the strongest workplace correlates of wellbeing and were robust to every specification. With scalar measurement invariance established, multi-group models showed that the association between employer-provided training and wellbeing was weaker among working-class than among middle-class employees (0.072 versus 0.130; Wald *p* = 0.001, jackknife *p* = 0.012), and the model explained 12.0% of working-class wellbeing compared with 31.1% among upper-class employees. The pattern suggests that equal provision of employer digital training may not yield equal benefit, although the class difference is consistent in direction rather than statistically conclusive across all specifications.

## 1. Introduction

Within two months of its launch in late 2022, ChatGPT had reached an estimated 100 million monthly users, the fastest uptake of a consumer application recorded up to that time (K. Hu, 2023), and in 2024 the European Union responded by enacting the world’s first comprehensive AI legislation. These developments signal that the transformation of work through Artificial Intelligence (AI) is no longer a distant prospect but a present reality whose consequences remain poorly understood. The speed of change brought about by AI has surpassed our comprehension of its implications. The issue is no longer whether there will be a transformation of working life through AI but rather how organizations must deal with the process of changes already taking place. For workers and organizations, the challenges are unique. AI is taking over tasks hitherto undertaken by employees, including applications in monitoring and appraising performance (Kellogg et al., 2020), and technology-driven concerns about job automation being felt in the workplace (Valtonen et al., 2023). However, organizations must make decisions about training and interventions with little guidance because the frameworks that describe how algorithmic systems reshape work (Kellogg et al., 2020; Parent-Rocheleau & Parker, 2022) have not yet been connected to a model of how organizational support translates into worker wellbeing, or for whom.

There is limited work in the literature that can enlighten us on the present question. Work on displacement effects, wage effects, and class effects of technological change (Acemoglu & Restrepo, 2022; Gil-Hernández et al., 2024) exists but treats workers merely as production factors whose experiences can simply be “inferred” from the data. Research on stress and burnout using approaches like the JD-R (Bakker & Demerouti, 2017) insufficiently considers the structurally mediated inequalities and subjective risks from technological change. The second set of literature on AI applications within organizations concentrates on how organizations apply the technology within the workplace (for example, algorithmic recruitment, autonomous performance management, and prediction-based workforce analytics) (Tambe et al., 2019; Duggan et al., 2020; Jatobá et al., 2023). This focus on the application potential of AI within organizational functions has led the literature away from a more pressing question that initially arose: What should the role of organizations be in protecting workers from the inevitable risks of AI technology in the organizational domain? Workplace training processes, support systems, and development processes could well end up helping employees navigate changes and risks posed by the application of AI technology in the organizational domain. However, there is a lack of literature on whether these processes work, whom these processes work for, and how these processes even work. The question addressed in this study is therefore specific: Does employer-provided training for digital technologies and AI benefit workers from different social classes equally?

This study aims to fill these research gaps by proposing a theoretical model that emphasizes the importance of training and support provided by the workplace as critical factors contributing to employees’ outcomes in AI-intensive environments. This study proceeds from two streams of theories, which until now have been largely separate. The JD-R theory offers a precise model of workplace processes, contrasting demands that lead to impaired health through the health impairment model, and resources that lead to positive outcomes through motivational gains (Bakker & Demerouti, 2007). We treat AI exposure as a potential modern job demand, one that may call for adaptive effort and generate uncertainty that could endanger employees’ security and autonomy (Scholze & Hecker, 2024), while leaving its demand character to be tested empirically. Digital skills and employer-provided digital training are viewed as resources that could offset job demands and are expected to be directly associated with better employee outcomes.

Despite this accuracy, however, the JD-R model lacks substantial grounding for specifying what outcomes should be of importance to researchers. For this, Sen’s capability approach provides an alternative framework for defining wellbeing. This approach locates wellbeing neither in subjective states nor in resource endowments, but in the substantial freedoms that individuals have to “live the life they have reason to value,” such that it reflects “functionings that they value” (Sen, 1999). In addition to this, it also states that “the same resource can be converted differently depending upon conversion factors,” while conversion factors, according to Robeyns (2017), “are personal and social characteristics that define how effectively individuals can convert resources into achieved functionings.” These conversion factors can be grouped according to social positions. Employees also possess varying backgrounds in terms of education, economic resources, social connections, and initial skills that can either convert AI exposure into an opportunity or an existential threat to flourishing. Through the combination of JD-R and capability assessment, it becomes possible to not only describe the effects of AI use among employees but also account for these effects across differing social classes in the workforce.

This framework develops a number of propositions that can be tested. AI exposure could be conceptualized as a demand that reduces capabilities for a given employee, both directly through autonomy and skill degradation (Wood et al., 2019), and indirectly through developing psychological strain. Digital skills and employer-provided digital training could be conceptualized as resources that protect wellbeing, whereas social class position indexes the conversion conditions under which such resources are used to ensure wellbeing. Most importantly, propositions could be specified to be a function of social class position in those given structural locations; employees experience AI in light of their conversion factors. Given identical AI exposure and identical employer interventions, employees in the working, middle and upper classes could nonetheless experience different wellbeing outcomes if their conversion factors differ. Equal provision would then not imply equal benefit.

We will examine these hypotheses using data from Special Eurobarometer 101.4, a cross-national survey of more than 8000 employed individuals across 27 European Union member states in April–May 2024 (European Commission, 2025). This survey includes indicators of exposure to AI in the workplace, digital skills, employer-provided training for digital technologies, appraisals of AI as a threat, as well as wellbeing factors that correspond to capability aspects. We used structural equation modeling (SEM) to estimate the proposed pathways, break down total effects into direct and indirect effects, as well as multi-group analysis to investigate class differences in all structural parameters.

The results have implications for research and workplace practice. Employer-provided digital training was, together with workers’ own digital skills, the strongest workplace correlate of wellbeing, whereas AI exposure itself was only weakly related to wellbeing and, once country clustering is taken into account, not reliably related to how threatening workers find AI. The central finding concerns who benefits. Although the measurement model was invariant across classes, the association between employer-provided training and wellbeing was weaker among working-class than among middle-class employees, a difference that held under country fixed effects and design-based jackknife standard errors but not under a country-clustered specification that treats the indicators as continuous, and the workplace model explained less than half as much of working-class wellbeing as of upper-class wellbeing. The pattern is consistent in direction across specifications but not statistically conclusive in all of them. It suggests, rather than establishes, that equal provision of employer training may not translate into equal benefit: workplace training is least strongly associated with wellbeing among the employees with the fewest conversion factors outside the workplace.

The paper is structured as follows: Section 2 provides a review of the literature on workplace AI, JD-R theory, and the capability approach. Following a discussion of their specificities, gaps in the literature are identified within which the intersectional approach lies. This allows us to segue into the theoretical model, following which Section 3 describes the materials and methods. Results are disclosed in Section 4, with implications for both theory and practices discussed in Section 5. Finally, Section 6 concludes the study.

## 2. Theory and Hypotheses

### 2.1. Workplace AI and the Human Centered Challenge

Algorithmic management has brought drastic changes to the nature of the employment relationship that begs for serious consideration and investigation from researchers and practitioners. According to Kellogg et al. (2020), there are six ways in which employees are controlled by algorithms. These are limiting choices through scheduling algorithms, suggesting what to do through nudges, tracking activity through continuous monitoring systems, rating productivity through automatic rating systems, supplanting judgment with determinations from algorithms, and rewarding or punishing workers on the basis of mathematical calculations. These methods ushered in a new era of labor control on the job that spread from their origin in the platform economy (Gandini, 2019). A Eurofound (2020) study shows that more than one quarter of European workplaces use data analytics technology to monitor employees. They also indicate that forty percent of large employers make use of technology. Yet, evidence from European companies shows workplace wellbeing drops due to algorithmic management practices (Kinowska & Sienkiewicz, 2023).

Disparity between administrative technology dependence and its adverse effects on employees puts organizations in a challenging situation. The prevailing focus in workplace research has been to adopt AI for improving organizational practices themselves, primarily for effective recruitment, performance management, and workforce analytics tasks (Tambe et al., 2019; Jatobá et al., 2023). On the other hand, there has been very little attention paid to the effects of AI on employees in general, in various functions within the organization. There is limited recognition that technology-related stress and employee wellbeing are crucial issues in the context of dealing with AI implementation in the workplace (Úbeda-García et al., 2025). However, as we shall propose, organizational functions dealing with training and development initiatives for employees could well counter the technology-related challenges employees face.

Current empirical research on AI and employees’ outcomes is still theoretically fragmented. Research has shown that algorithms have a design role with regard to work and influence changes in job characteristics with regard to autonomy and monitoring (Parent-Rocheleau & Parker, 2022; Parent-Rocheleau et al., 2024). The use of AI raises job stress for employees who believe they have limited adaptability abilities (Kim & Lee, 2024), while for employees working in the gig economy, there is an “autonomy paradox” with regard to flexibility and algorithmic control as defined within the study made by Wood et al. in 2019. In this area, there is a need for a guiding model connecting the mechanisms triggered in employees’ wellbeing because of AI use and specifying whose wellbeing is a priority and where organizational interventions could help. The model will be developed using two theories: the job demands–resources (JD-R) model originating from organizational psychology and Sen’s capability approach originating in welfare economics.

### 2.2. Job Demands–Resource (JD-R) Framework

The job demands–resources theory is the mechanistic basis for our analysis. Bakker and Demerouti (2007, 2017) argue that all work characteristics are either demands or resources, each impacting through a different process. More recent work has generalized the theory to encompass modern challenges such as technological change and times of crisis (Bakker et al., 2023; Demerouti & Bakker, 2023) and has combined it with the theory of planned behavior to explain when employees support the digital-intelligence transformation of their organizations (T.-J. Wu et al., 2025). Job demands are work aspects that place a chronic or sustained demand on efforts, with associated costs at the biological or psychological level. Job resources are work aspects that help to achieve goals, reduce or prevent demands, or contribute to growth and development goals. Definitively, rather than summing to a net job quality, job demands and job resources each utilize different processes that deserve separate consideration.

The process of health impairment explains how pressure leads to strain. The repeated presence of high pressure drains workers’ energy levels, beginning a process that could lead to exhaustion and ultimately burnout. The original study on the theory of job demands and resources has empirically defined this process, with strategic reviews validating this process in the workplace (Galanakis & Tsitouri, 2022; Demerouti et al., 2001). The motivational process explains how resources lead to engagement because of predefined psychological needs for autonomy, competence, and relatedness. The two processes tend to remain relatively independent. This has significant practical implications. An effective strategy to reduce demands and a strategy to build resources will both lead to better results. Both happen for different reasons.

We define “workplace AI exposure” as a type of current job demand. This is because demands have the following essential features: they involve adaptive effort, entail psychological costs, and generate conditions leading to resource depletion. Workers need to get used to operating AI systems without proper training, conform to algorithmic instructions that can contradict professional standards, and negotiate with uncertainty about their personal relevance. Algorithmic monitoring entails what Newlands (2021) calls spatial abstraction. Here, workers get reduced to data points, as much as their embodied practices get effaced. Algorithmic monitoring for job performance exerts pressure that differs from that of human monitoring, in that algorithms lack discretion and tend to hold unknown standards. There is evidence that employees who are aware of AI in their workplace report more depressive symptoms, an association that runs through emotional exhaustion and is weaker when perceived organizational support is high (Xu et al., 2023), while robot exposure is linked to job insecurity and maladaptive workplace attitudes (Yam et al., 2023). Working with robots and AI is not uniformly harmful; however, Duan et al. (2026a) find that human–robot collaboration is associated with more organizational citizenship behavior through employees’ identification with the collaborative robot, with emotional exhaustion as a competing pathway, and longitudinal evidence from Germany finds no sizeable negative effect of occupational AI exposure on workers’ wellbeing or mental health, although workers who themselves use AI tools report somewhat lower life and job satisfaction (Giuntella et al., 2025). We also note that exposure to AI systems is not the same as experienced work pressure: the survey items used here record how often algorithmic systems perform management activities in the respondent’s workplace, not the effort or strain these systems generate. We therefore treat AI exposure as an indicator of a potential job demand whose demand character is itself an empirical question, and we return to this point when interpreting the results.

Digital skills and employer-provided digital training are the resources at the center of our model. Digital skills are a personal resource that is critical for effective engagement with AI systems and form the human capital that guards against job insecurity. Employer-provided digital training, measured here through whether the employer provides the tools or training needed to work with recent digital technologies, including AI, is an organizational resource that signals the organization’s commitment while enhancing employees’ adaptability. Investments in training and development influence employee outcomes through both competence and commitment, with voluntary participation and evaluation strengthening these effects (Sung & Choi, 2018), and in the OECD AI surveys, workers whose employers provided training for AI reported more positive effects of AI on their work (Lane et al., 2023). From a practical perspective, employer-provided training is the variable that falls most directly within the organization’s control: organizations cannot directly change the skills their employees bring, but they can invest in the programs that build them.

### 2.3. The Capability Approach: Conversion Factors and Heterogeneity

The JD-R model spells out mechanisms but is brief about specifying what counts and why, as an outcome. In this study, however, Sen’s capability approach (Sen, 1999) will be applied instead, defining wellbeing instead of utility and resources by the concrete freedoms that persons enjoy in living a life they judge as valuable. Capabilities are the real freedoms a person has to achieve valued functionings, that is, the “beings and doings” (such as being financially secure or being satisfied with one’s life) that make up a flourishing life (Robeyns, 2005, 2017). Capabilities for employees include material security, using judgment and skill, control over work, recognition, and assurance in communal life. New theoretical developments have specifically applied capability theory in job settings, with quality-of-work frameworks conceptualized based on what can actually be accomplished by employees, according to Stephens (2023).

The capability approach also offers the theoretical grounding to justify this expectation of diversity. The same resources lead to different capability levels based on conversion factors, the personal and social features that influence the degree to which individuals can transform resources into accomplished capabilities (Robeyns, 2017). A skilled worker empowered with educational and financial assets has conversion factors that enable them to make use of AI in a capability-enhancing manner. Another worker without conversion factors might face the same amount of AI contact in a capability-detracting manner. Sen (1997) argued that unemployment and precarious employment impose costs beyond lost income, including psychological distress, the loss of skills through disuse, and social exclusion, so that the same labor market event produces systematically different levels of capability disadvantage for differently placed people.

Conversion factors are grouped according to social class. Upper-class employees are equipped with educational credentials that are a basis for skill learning, social connections that offer information and access, financial assets that enable investment in skill advancement, and bargaining power to secure beneficial terms of implementation. As Wolff and de-Shalit (2007) posit, working-class employees are deprived of these factors and are thus more susceptible to negative consequences, facing “corrosive disadvantage” that hinders functioning in various aspects of life because of deprivation in one area. Current evidence affirms that technological change has task mechanisms that differ for each social class, leading to differentiated outcomes in European labor markets (Gil-Hernández et al., 2024). The middle class falls in between: it holds some of these conversion factors, although to a lesser extent than the upper class, while the routine cognitive tasks that characterize many middle-class jobs are increasingly exposed to automation through AI applications. We stress that self-placed social class does not measure these conversion factors directly. It is used in this study as a summary indicator of the structural position with which education, economic resources, social networks and bargaining power tend to covary, that is, as a proxy for differences in conversion conditions rather than as a conversion factor in itself.

We draw on the capability approach in two distinct ways, and it is important to be precise about both. First, as an evaluative framework, it specifies what should count as the outcome: not resource holdings and not utility alone, but the freedoms and achievements that people have reason to value. Our outcome measure does not capture the capability set; it combines an achieved functioning in the material domain (being able to pay one’s bills) with two evaluations of life as a whole. We therefore call it capability-relevant wellbeing to signal that it captures part of what the approach values without claiming to measure capabilities. Including financial security alongside life satisfaction is consistent with the JD-R motivational process, in which resources that raise employability and earning capacity are expected to protect workers’ material position, and with the capability tradition’s insistence that material security is a central functioning (Sen, 1999; Stephens, 2023). Second, as a heuristic device, the approach supplies the notion of conversion factors, from which we derive the expectation that the same workplace resource yields different wellbeing gains across social positions. Read in this way, the capability approach frames the outcome and the heterogeneity question; it does not supply a measurement model of capabilities, and we do not claim that it does.

### 2.4. An Integrated Framework for Understanding AI and Worker Wellbeing

Integration of the different perspectives above results in a framework that views organizational interventions as being central to the employee outcome process within the context of the AI transition process. Exposure to AI is treated as a potential job demand that may be associated with lower capability-relevant wellbeing through several mechanisms. The first pathway is indirect and runs through strain: exposure to AI is expected to generate psychological strain, which in turn lowers wellbeing. In this study, the strain pathway is captured through threat appraisal, that is, workers’ appraisal of AI as a threat to their jobs, job security and workplace relationships. We treat threat appraisal as the cognitive component of the strain pathway rather than as a measure of experienced strain itself.

The logic of the combined model can be stated compactly. Workplace AI exposure is the (potential) job demand; digital skills (a personal resource) and employer-provided digital training (an organizational resource) are the job resources; threat appraisal is the cognitive component of the strain pathway; capability-relevant wellbeing is the outcome; and social class is the conversion condition that is expected to moderate how resources translate into wellbeing. The JD-R model supplies the mechanisms, the health impairment and motivational processes, but it is silent about who benefits from resources. The capability approach adds two elements that the JD-R model lacks: an evaluative criterion for the outcome (freedoms to achieve valued functionings rather than utility or resource holdings) and, through conversion factors, a principled reason to expect that the same organizational resource yields different wellbeing gains across social positions. Without the second element, class differences in the returns to training would be an unexplained empirical curiosity; with it, they become a testable prediction.

Digital skills and employer-provided digital training enter the model as resources in the JD-R sense: they help workers meet the demands of AI-mediated work and satisfy needs for competence and security. Whether these resources are used to ensure wellbeing, and how efficiently, is expected to depend on conversion conditions that lie largely outside the workplace, which we index with social class. Employer-provided training deserves particular attention because it is the resource most directly under organizational control. Recent reviews position employee wellbeing as a central goal of AI integration rather than a by-product of it (Panda et al., 2025; Úbeda-García et al., 2025); survey evidence indicates that training goes together with more favorable worker-reported outcomes of AI (Lane et al., 2023); and collaboration with AI has been found to raise hotel employees’ quality of work life when it serves both their basic and their growth needs (Duan et al., 2026b). The provision of training by the employer is therefore expected to be a robust correlate of employees’ wellbeing, which underlines the importance of organizational development functions during technological change.

Social class is therefore expected to moderate the relationships in the model, most clearly the use of resources to ensure wellbeing because each position in the social structure carries a distinct profile of conversion factors. The same exposure to AI systems and the same organizational support may thus be associated with different wellbeing outcomes depending on social position. This will have direct implications for intervention targeting, as the optimal allocation of resources depends on knowing what groups of employees will respond best.

### 2.5. Hypotheses

This integrated framework develops the following hypotheses. Because our outcome measure captures capability-relevant wellbeing (financial security and life satisfaction) rather than the full capability set, we refer to it as capability-relevant wellbeing, shortened to wellbeing in the hypotheses, tables, and figures.

For job demand, the health impairment process predicts that demands generate strain. Technostress research shows that intensive technology use is associated with techno-insecurity and strain (Tarafdar et al., 2007; Ragu-Nathan et al., 2008), and exposure to robots and AI has been linked to job insecurity (Yam et al., 2023) and to AI-related job stress (Kim & Lee, 2024). Employees working with AI systems must also devote adaptive effort to them. We therefore hypothesize the following:

**H1a.**  *AI exposure is positively associated with threat appraisal.*

Demands may also be associated with wellbeing directly, through non-strain mechanisms such as loss of autonomy and reduced skill use under algorithmic management (Wood et al., 2019; Parent-Rocheleau & Parker, 2022; Felix et al., 2023). Accordingly,

**H1b.**  *AI exposure is negatively associated with wellbeing, net of indirect effects through threat appraisal.*

Concerning resources, both JD-R theory and the capability approach expect resources to be positively associated with outcomes. Digital skills facilitate effective use of AI and are associated with lower techno-insecurity (Ragu-Nathan et al., 2008) and with better labor market positions. Employer-provided training builds adaptability and signals organizational commitment (Sung & Choi, 2018); in the OECD AI surveys of workers in AI-using firms, employer-provided training went together with more positive reported effects of AI on performance, working conditions, and job satisfaction (Lane et al., 2023).

We expect the following:

**H2a.**  *Digital skills are positively related to wellbeing.*

**H2b.**  *Employer-provided digital training is positively related to wellbeing.*

Resources may also be associated with lower threat appraisal. Workers who feel competent with digital technologies, and whose employers equip them to work with these technologies, have less reason to appraise AI as a threat to their jobs (Ragu-Nathan et al., 2008; Kim & Lee, 2024). We note that the model specifies direct paths from the resources to threat appraisal rather than demand × resource interactions. H3a and H3b are therefore hypotheses about resource–appraisal associations, not buffering hypotheses in the strict JD-R sense, in which resources weaken the effect of demands on strain (Bakker & Demerouti, 2017). The interaction form of buffering is examined in a supplementary analysis (Section 4.6).

**H3a.**  *Digital skills are negatively associated with threat appraisal.*

**H3b.**  *Employer-provided digital training is negatively associated with threat appraisal.*

The health impairment process proposes strain as a mediator between demands and outcomes (Bakker & Demerouti, 2017). AI exposure may therefore be associated with lower wellbeing partly through the following threat appraisal:

**H4.**  *Threat appraisal partially mediates the relationship between AI exposure and wellbeing.*

Threat appraisal should itself be associated with lower wellbeing, since perceived job insecurity is a well-documented correlate of lower life satisfaction and material worry (Sen, 1997; Yam et al., 2023).

**H5.**  *Threat appraisal is negatively associated with wellbeing.*

Finally, the capability approach leads us to expect heterogeneity across social classes because conversion factor profiles differ by social position. The arguments in Section 2.3 point in more than one direction. The conversion-factor argument identifies working-class employees, who hold the fewest complementary assets, as the group least able to use workplace resources to ensure wellbeing (Wolff & de-Shalit, 2007), whereas the literature on the automation of routine cognitive tasks suggests that middle-class employees may be the most exposed to AI as a demand (Gil-Hernández et al., 2024). Because these arguments concern different paths and would require assumptions beyond the scope of this study to combine into class-specific directional predictions, we state the class hypothesis as an open hypothesis of heterogeneity. In the original design of the study, we expected the middle class to be the most sensitive group; we retain this expectation as a secondary, exploratory question and note in Section 5 where the results depart from it.

**Class Moderation (CM):** The relationships posited in H1 to H5 differ across social classes; in particular, the use of workplace resources (digital skills and employer-provided digital training) to ensure wellbeing differs by class.

## 3. Materials and Methods

### 3.1. The Sample

We use the Special Eurobarometer 101.4 data, which is a cross-sectional study performed by the European Commission in April and May 2024 and concentrates on digitalization and AI at the workplace (European Commission, 2025). The Eurobarometer study uses a multi-stage probability technique of conducting face-to-face interviews and covers the adult population of all 27 European Union countries (28 Geographical units, with Germany being divided into east and west). The total sample size is 26,404 participants, out of which our study selected the employed section of respondents pertaining only to our study questions.

The analytical sample was constructed in six steps (Online Supplementary Materials Table S1). First, because AI exposure at work and employer-provided training are defined only for people in work, we retained the 13,883 respondents who were currently employed. Second, we required complete data on the 16 indicators of the four latent constructs (n = 9664). Third, the employer training item (QB3) was asked only of respondents who work for an employer, so the 1379 self-employed respondents, who have no employer, could not be included, and 132 employees who answered “not applicable” or “don’t know” to this item were excluded (n = 8153). Fourth, 38 respondents without a valid self-placed social class were excluded, giving 8115 respondents for the measurement model. Finally, listwise deletion on the seven control variables (firm size, 31; gender, 16; education, 2; internet use, 1) left 8065 respondents for all structural models. Online Supplementary Materials Table S1b compares the retained respondents with the 1599 employed respondents who had complete indicator data but were excluded at steps three to five. The excluded group consists mainly of the self-employed and is accordingly older, more often male and concentrated in very small firms, with lower workplace AI exposure; it does not differ from the retained sample in wellbeing (Cohen’s d = 0.05, *p* = 0.11) and differs only slightly in digital skills (d = 0.11) and threat appraisal (d = −0.08). The analytical sample should therefore be read as a sample of employees rather than of all working people. The main results are replicated on the enlarged sample that includes the self-employed when the training item is dropped from the model (Section 4.5).

Table S1 shows that in multiple group analysis, the sample has been stratified for social class and classified into three groups: working class (n = 1454; 18.0%), middle class (n = 5788; 71.8%), and upper class (n = 823; 10.2%) in the structural-model sample. These groups surpass the minimum required levels for the multiple group structural equation modeling (SEM) analysis (Kline, 2023).

### 3.2. Measures

The theoretical model needs to operationalize AI exposure as a potential job demand, digital skills and employer-provided digital training as resources, threat appraisal as a mediator, and wellbeing as the outcome. All constructs with their indicators are presented in Table 1 with their scales and reliability statistics; indicator wording and descriptive statistics are reported in Online Supplementary Materials Table S2.

AI exposure measures the presence of AI and algorithmic systems in the respondent’s work environment. Six items (QB7) ask whether the following activities have been performed by digital technologies, including AI, in the respondent’s current or most recent workplace: hiring workers, allocating tasks to workers, managing worktime schedules, monitoring workers’ activities, assessing workers’ performance (including imposing sanctions or attributing rewards), and enforcing safety measures. Responses were coded so that higher values indicate more exposure (1 = no, never; 4 = yes, all the time). Internal consistency is high (α = 0.91). As noted in Section 2.2, the items record the frequency of algorithmic management practices, not the effort or pressure they generate; the construct is therefore an indicator of a potential job demand rather than a measure of experienced demand.

Digital skills are a personal resource that facilitates effective functioning in the context of AI systems. The four items (QB2) ask whether respondents consider themselves sufficiently skilled in the use of the most recent digital technologies, including AI, in their daily life, to do their job, to find a job or change jobs within the next twelve months, and to benefit from digital and online learning opportunities (1 = totally disagree to 4 = totally agree after reverse coding; α = 0.90). From a practical perspective, digital skills are the human capital that training and development initiatives aim to build.

Employer-provided digital training is the organizational resource. It is measured with a single item (QB3) asking respondents to what extent they agree or disagree that “your employer provides you with the necessary tools or training to work effectively with the most recent digital technologies, including Artificial Intelligence” (1 = totally disagree to 4 = totally agree after reverse coding; “not applicable” and “don’t know” treated as missing). We label the construct by what the item measures. It captures one specific form of organizational support, the provision of tools and training for digital technologies, rather than perceived organizational support in the broader sense of the term (Eisenberger et al., 1986), and the label should not claim more than the measure. The item nevertheless represents the training and development function that our theoretical model identifies as the organizational lever most directly under management control. Single-item measures are acceptable when the construct is concrete and unambiguous, and they have been shown to perform comparably to multi-item scales in structural equation models under these conditions (Hayduk & Littvay, 2012); the limitation this entails is discussed in Section 5.5.

Threat appraisal represents technology-related concerns and acts as the mediating variable in the health impairment pathway. We label this construct threat appraisal rather than psychological strain because its items record agreement with statements about AI as a threat to jobs and workplace relationships, that is, an appraisal of the technology rather than a report of experienced strain. The three items (QB6) are “due to the use of robots and artificial intelligence, more jobs will disappear than new jobs will be created”, “robots and artificial intelligence steal people’s jobs” and “robots and artificial intelligence have a negative impact on communication between colleagues” (1 = totally disagree to 4 = totally agree after reverse coding; α = 0.76). A fourth item on AI increasing the pace of work was dropped to improve model fit.

Capability-relevant wellbeing is the outcome. Three items are used: whether the respondent had difficulties paying bills at the end of the month during the last twelve months (D60; 1 = most of the time, 3 = almost never or never), satisfaction with the life they lead (D70), and satisfaction with their daily life (D70a; 1 = not at all satisfied to 4 = very satisfied). The first item records an achieved functioning in the material domain; the other two are global evaluations of life. As explained in Section 2.3, the combination reflects the capability approach’s insistence that both material conditions and a person’s own evaluation of their life matter, but it does not measure capabilities as such (α = 0.77).

We also measured social class on the basis of self-classification. This allowed the respondent to describe her/his family as belonging to the working class, lower middle class, middle class, upper middle class, or higher class. We combined these into three groups while maintaining the theoretically informed distinction between the working-class, middle-class, and upper-class positions. The subjective class classification taps into how people perceive themselves within a structural context. Subjective class position has been argued to carry implications for the conversion factors that matter in the capability approach (Robeyns, 2017); as noted in Section 2.3, it is used here as a proxy for differences in conversion conditions, not as a direct measure of conversion factors.

Seven control variables are incorporated for the prediction of wellbeing, namely age, gender, level of educational attainment, size of firm, internet use, level of urbanization, and income categories of countries. These seven variables, with their definition and measurements, are listed in Table 1 (Panel B), and their definitions and construction are detailed in Online Supplementary Materials Table S5.

### 3.3. Analytical Strategy

**Figure 1:**
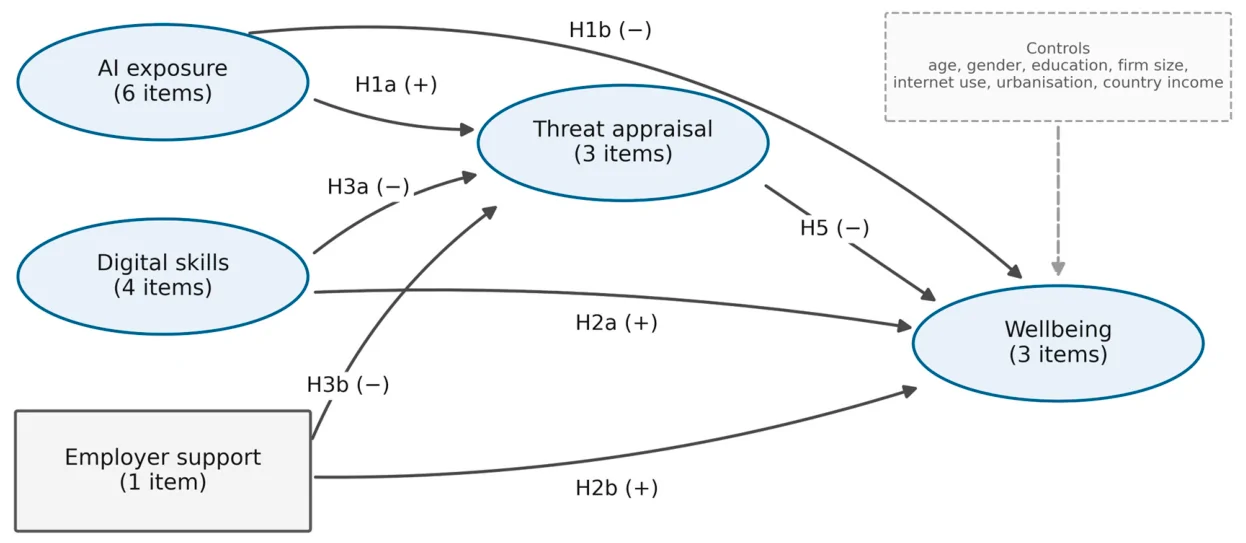
**Hypothesized structural model. *Note.*** Ellipses denote latent constructs measured with ordinal indicators (number of items in parentheses); the rectangle denotes a single observed indicator. “Employer support” means employer-provided digital training. Signs in parentheses give the hypothesized direction of each path. H4, the indirect effect of AI exposure on wellbeing through threat appraisal, is the product of H1a and H5 and is not drawn separately. Control variables (dashed box) predict wellbeing only. Class moderation is tested by estimating the same model for working, middle, and upper-class workers in a multi-group framework with all structural paths freely estimated.

We propose testing the model relationships using structural equation modeling and validate our results with machine learning techniques. The hypothesized model structure is shown in Figure 1.

SEM was chosen based on the following reasons. First, SEM can estimate the measurement component and the structural component simultaneously by considering the measurement error in the constructs (Bollen, 1989). Second, SEM can easily test the mediated effect by estimating the indirect effect directly (Hayes, 2018). Lastly, the multi-group SEM can effectively compare the structural parameters among the social classes (Vandenberg & Lance, 2000).

All analyses were performed using lavaan and R (Rosseel, 2012). Due to the use of three- to four-point rating scales for the ordinal indicators, the model was estimated using the weighted least squares mean and variance (WLSMV) estimator, which is particularly suited for modeling categorical indicators through polychoric correlations (Li, 2016). For model fit evaluation, the following indices were considered according to typical guidelines (L. Hu & Bentler, 1999): the comparative fit index (CFI), the Tucker–Lewis index (TLI), the root mean squared error approximation (RMSEA), and the standardized root mean squared residual (SRMR). To address potential common method bias inherent in single-source survey data, we assessed discriminant validity through the measurement model and examined whether method variance inflated correlations beyond theoretically expected magnitudes (Podsakoff et al., 2003).

We adopted the two-step procedure as advocated by Anderson and Gerbing (1988). In the first step, the measurement model was verified by confirmatory factor analysis (CFA) for convergent and discriminant validity. In the second step, we specified the theoretical relationships from AI exposure and the two resources to threat appraisal; from AI exposure, the resources, and threat appraisal to wellbeing; and from the controls to wellbeing. Effects in mediation analyses were calculated by multiplying path components, and significance was assessed using bootstrapping methods for confidence interval evaluation.

Multi-group analysis followed the sequential procedure for measurement invariance (Vandenberg & Lance, 2000), adapted to ordinal indicators following H. Wu and Estabrook (2016). A configural model was followed by models constraining thresholds, then thresholds and loadings (metric invariance), and then thresholds, loadings, and intercepts (scalar invariance). Invariance was judged with ΔCFI < 0.01 and ΔRMSEA < 0.015 (F. F. Chen, 2007) because scaled χ^2^ difference tests are sensitive to trivial misfit at this sample size. After invariance was established, the structural model was estimated with all structural paths freely estimated in each class. Cross-class equality of each path was tested with Wald tests (2 df) and with scaled χ^2^ difference tests comparing the free model with a model constraining that path to equality (Satorra, 2000); an omnibus test constrained all seven structural paths simultaneously.

Because respondents are nested in 28 country samples, we examined the sensitivity of the pooled and multi-group results to country heterogeneity in three ways (Section 4.5). First, we replaced the country income group control with country fixed effects (27 dummy variables) in the WLSMV models, which absorb all country-level differences in the level of wellbeing. Second, we re-estimated the models with robust maximum likelihood (MLR) and standard errors clustered by country; this is the only design-based option available for these models in lavaan, but it treats the three- and four-category indicators as continuous. Third, we ran a delete-one-country jackknife of the WLSMV multi-group model, refitting it 28 times with one country sample omitted each time, which yields design-based standard errors for the cross-class contrasts while retaining the ordinal estimator. We also re-estimated the pooled model with the survey weights. Finally, because H3a and H3b concern direct resource–appraisal paths rather than buffering interactions, we tested the interaction form of buffering in a supplementary analysis using composite scores (Section 4.6).

To complement the parametric analysis of SEM, machine learning (ML) validation via Random Forest (Breiman, 2001) and XGBoost (T. Chen & Guestrin, 2016) using SHAP values (Lundberg & Lee, 2017) was conducted on the same 8065 respondents, using composite scores for the constructs (item means; the wellbeing indicators were standardized before averaging because they use different response scales). This procedure examines if rankings on variable importances obtained from prediction optimization algorithms agree on SEM coefficient estimates and if dependence plots of SHAP on variable importances indicate nonlinearities unspecified in the SEM model. See Section 4 for ML and SEM results.

## 4. Results

### 4.1. Measurement Model

The measurement model with four latent variables fitted the data well (N = 8115), with CFI = 0.997, TLI = 0.997, RMSEA = 0.040, and SRMR = 0.036. The factor loadings were significant and above 0.50. The loadings for AI exposure ranged from 0.799 to 0.875, digital skills from 0.854 to 0.880, threat appraisal from 0.636 to 0.858, and wellbeing from 0.514 to 0.976. Composite reliability for the four variables was above 0.80. Lastly, the results support discriminant validity, and this was established with the Fornell–Larcker criterion. These findings indicate reliable measurement of the latent variables and the distinctness of the theoretical concepts. The results for the measurement model, including the loadings, are shown in Online Supplementary Materials Tables S3 and S4.

Measurement invariance across social classes was then tested on the structural-model sample (N = 8065). As Table 2 (Panel B) shows, constraining thresholds (ΔCFI = −0.0011), thresholds and loadings (ΔCFI = 0.0001), and thresholds, loadings and intercepts (ΔCFI = −0.0010) left fit essentially unchanged, and all ΔRMSEA values were below 0.002 in absolute value. Full scalar invariance therefore holds, so the latent constructs are measured in the same way in each class and the class comparisons below are meaningful.

### 4.2. Structural Model

The fully specified SEM (N = 8065) showed an acceptable fit: CFI = 0.967, TLI = 0.982, RMSEA = 0.082, SRMR = 0.032. The model accounted for 22.9% of the variance in wellbeing, a relatively high amount for cross-sectional studies of subjective wellbeing. The results are reported in Table 3. The path diagram showing coefficients can be found in the Online Supplementary Materials, Figure S1. The findings include results that support and results that contradict the theoretical expectations.

Turning first to AI exposure, we hypothesized that workers more exposed to workplace AI would appraise AI as more threatening (H1a) and report lower wellbeing (H1b). H1b was supported: AI exposure was negatively associated with wellbeing (β = −0.073, *p* < 0.001), an association consistent with, though not evidence of, losses of autonomy or skill use. Two qualifications apply. First, the zero-order latent correlation between AI exposure and wellbeing is close to zero (r = 0.008, *p* = 0.550; Online Supplementary Materials Table S4). The negative association appears only net of digital skills, employer-provided digital training, and the controls because AI exposure is positively correlated with digital skills (r = 0.185), which are themselves associated with higher wellbeing. Second, the association is small. H1a was not supported: AI exposure was negatively rather than positively associated with threat appraisal (β = −0.037, *p* = 0.005), so workers with more exposure found AI slightly less threatening. We refer to this as an AI familiarity association, but it is a secondary finding. AI exposure, digital skills, and employer-provided training together explain 0.2% of the variance in threat appraisal (R^2^ = 0.002), and the association does not survive design-based standard errors (Section 4.5). Threat appraisal is therefore essentially independent of direct workplace experience with the technology, and none of our conclusions rest on this association.

The resource hypotheses produced the results of most relevance for organizations. Digital skills were positively associated with wellbeing (β = 0.189, *p* < 0.001), supporting H2a and consistent with the protective role of personal resources. Employer-provided digital training had the largest coefficient among the theoretical constructs (β = 0.209, *p* < 0.001), comparable in size to country income group (β = 0.208), supporting H2b. Both coefficients are moderate in absolute terms, but they are the strongest workplace correlates of wellbeing in the model and are robust across all specifications reported in Section 4.5. The result gives a numerical anchor to practitioners’ claims about the role of employee skill development during the adoption of AI, and it places the training function close to the center of that process rather than at its periphery.

Results for the resource–appraisal hypotheses were mixed. Digital skills were not associated with threat appraisal (β = 0.004, *p* = 0.799), so H3a was not supported. Employer-provided digital training had a small negative association with threat appraisal (β = −0.028, *p* = 0.023), supporting H3b under conventional standard errors, although the association does not survive design-based inference (Section 4.5). These results indicate that, in this model, resources are associated with wellbeing mostly directly rather than through lower threat appraisal. The interaction form of buffering is examined in Section 4.6.

Threat appraisal was negatively associated with wellbeing (β = −0.081, *p* < 0.001), supporting H5. Employees who appraised AI as a threat reported lower financial security and life satisfaction, indicating that threat appraisal is relevant to wellbeing even though, as shown above, it is barely related to AI exposure itself. Under design-based standard errors, this association lies at the margin of conventional significance (*p* = 0.045 to 0.052; Section 4.5).

### 4.3. Mediation Analysis

To investigate whether threat appraisal mediated the relationship between AI exposure and wellbeing (H4), we calculated the indirect effects with bootstrapped confidence intervals (Table 3, Panel C; class-specific estimates in Online Supplementary Materials Table S6). The indirect effect was statistically significant but operated in an unexpected direction (indirect effect = 0.003, *p* = 0.011). Because greater exposure is associated with slightly lower threat appraisal, and lower threat appraisal is associated with higher wellbeing, the product term is small and positive. The direct association, which is larger and negative, dominates this small positive indirect component, giving a negative total association (−0.070, *p* < 0.001). H4 is therefore not supported.

The association of employer-provided digital training with wellbeing was both direct (β = 0.209) and, to a very small extent, indirect (0.002, *p* = 0.032), with the largest total association among the theoretical predictors (0.212, *p* < 0.001). The indirect component is negligible in size; the association runs almost entirely through the direct path.

### 4.4. Class Heterogeneity

One of the main contributions of this research is the examination of how the associations in the model differ by social class. After establishing full scalar invariance (Table 2, Panel B), we estimated the multi-group model with all structural paths free in each class and tested whether each path differed across classes.

**Figure 2:**
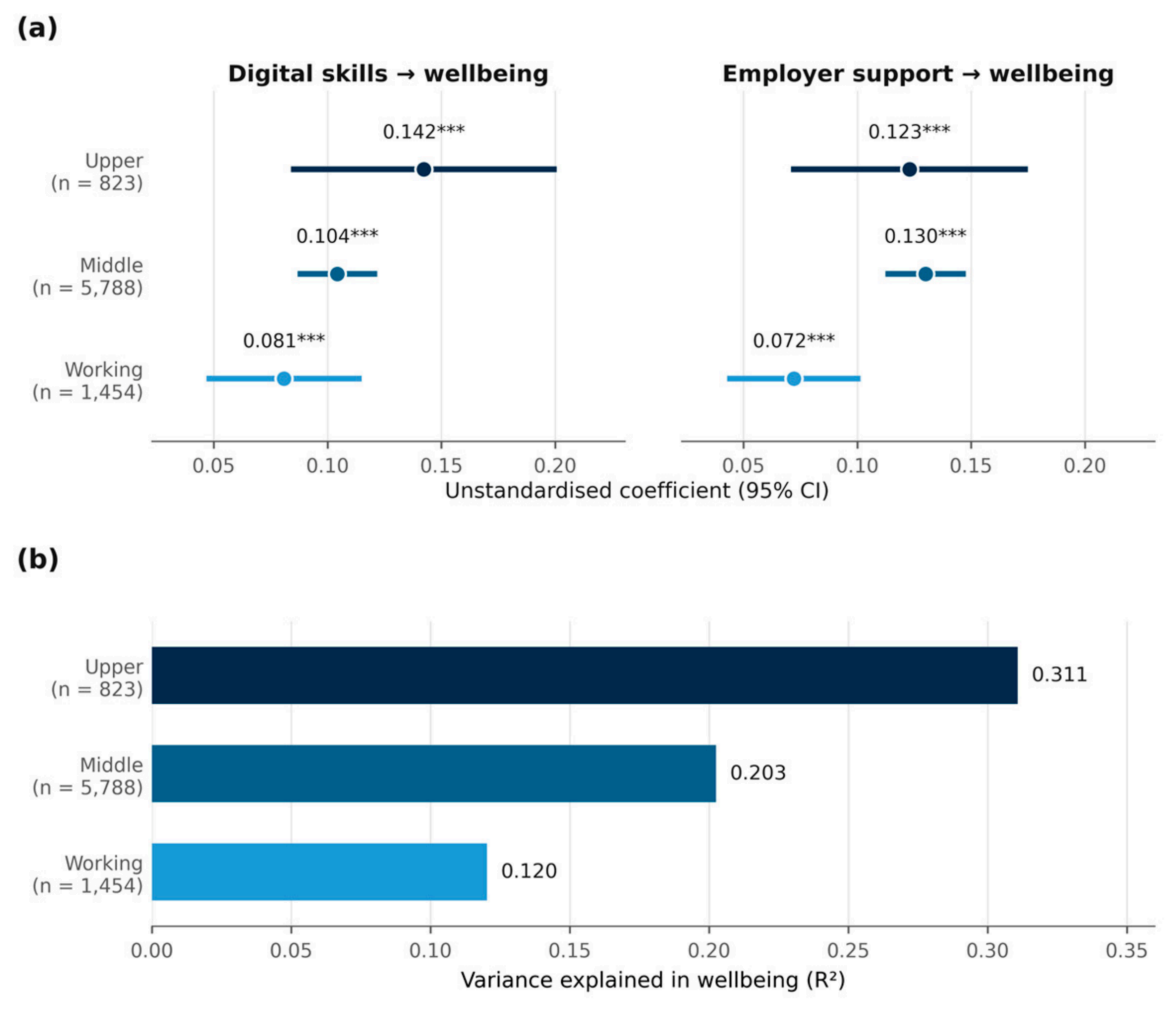
**Social class and the use of workplace resources to ensure wellbeing. *Note.*** N = 8065 (working class = 1454; middle class = 5788; upper class = 823). Both panels come from a multi-group structural equation model (WLSMV estimator, ordinal indicators) in which all structural paths were freely estimated across social classes, after scalar measurement invariance had been established (Table 2). (**a**) Unstandardized coefficients for the two resource → wellbeing paths; horizontal bars are 95% confidence intervals. Employer Support (Employer digital training) → wellbeing differs across classes, Wald χ^2^(2) = 11.08, *p* = 0.004 (scaled χ^2^ difference test: χ^2^(2) = 10.55, *p* = 0.005); the contrast is carried by middle versus working class, *p* = 0.0009 (middle versus upper *p* = 0.800; upper versus working *p* = 0.096). Digital skills → wellbeing does not differ significantly across classes, Wald χ^2^(2) = 3.37, *p* = 0.186. (**b**) Variance explained (R^2^) in wellbeing by the same model: 12.0% for the working class, 20.3% for the middle class and 31.1% for the upper class. Upper-class estimates should be interpreted with caution: the solution for that group contains a Heywood case (negative residual variance on one wellbeing indicator) that arises from sparse response categories. *** *p* < 0.001.

Table 4 and Figure 2 show that class heterogeneity is concentrated in how resources are used to ensure wellbeing rather than in the association with AI exposure. Constraining all seven structural paths to equality across classes significantly worsened fit (Δχ^2^(14) = 33.36, *p* = 0.003). The clearest difference concerns employer-provided digital training. The unstandardized association between employer-provided digital training and wellbeing was 0.072 among working-class employees, 0.130 among middle-class employees, and 0.123 among upper-class employees, and equality across the three classes was rejected (Wald χ^2^(2) = 11.08, *p* = 0.004; scaled χ^2^ difference test: χ^2^(2) = 10.55, *p* = 0.005). The difference lies between the working class and the other classes: the middle-versus-working contrast was significant (*p* = 0.0009), the upper-versus-working contrast pointed in the same direction but was not significant (*p* = 0.096), and the middle and upper classes did not differ (*p* = 0.800). The same level of employer-provided digital training is thus associated with a wellbeing difference among working-class employees little more than half as large as among middle-class employees. Section 4.5 examines whether this contrast survives country heterogeneity and design-based inference.

Digital skills showed an ordering of the same kind (0.081, 0.104, and 0.142 for the working, middle, and upper classes), but the difference was not statistically significant (Wald χ^2^(2) = 3.37, *p* = 0.186). The direct association between AI exposure and wellbeing did not differ across classes (χ^2^(2) = 1.57, *p* = 0.455), nor did the association between threat appraisal and wellbeing (χ^2^(2) = 0.12, *p* = 0.940). Two paths to threat appraisal did differ: AI exposure was negatively associated with threat appraisal among working- and middle-class employees but weakly positively among upper-class employees (χ^2^(2) = 9.17, *p* = 0.010), and digital skills reduced threat appraisal only among upper-class employees (χ^2^(2) = 15.17, *p* < 0.001). Because the model explains less than 3% of the variance in threat appraisal in every class, because the differences are carried by the upper-class group whose solution contains a Heywood case, and because they do not survive design-based inference (Section 4.5), we do not interpret them substantively.

The explanatory power of the model followed the same ordering. The workplace model accounted for 12.0% of the variance in working-class wellbeing, 20.3% in middle-class wellbeing and 31.1% in upper-class wellbeing (Figure 2b). The model therefore accounts for a smaller share of the variance in working-class wellbeing. This indicates only that the variables included in the model explain less of this group’s wellbeing, which omitted factors account for the remainder; for example, housing security, material constraints, or non-work circumstances cannot be determined from the present analysis and are questions for future research.

Estimates for the upper class should be read with caution. The upper-class solution contains a Heywood case (a small negative residual variance on one wellbeing indicator, with a standardized loading of 1.005) that arises from sparse response categories in the smallest group. We therefore base the class argument on the working-versus-middle contrast, which does not depend on the upper-class estimates.

Section 4.5 reports robustness checks for the pooled model and for this class contrast.

### 4.5. Robustness to Country Heterogeneity and Sample Composition

Because respondents are nested in 28 country samples, the pooled model was re-estimated in several ways (Online Supplementary Materials Table S12). Replacing the country income group control with 27 country fixed effects left every path unchanged in sign and significance (employer-provided digital training β = 0.181, digital skills β = 0.153, AI exposure β = −0.046, threat appraisal β = −0.054, all *p* < 0.001; AI exposure → threat appraisal β = −0.055, *p* < 0.001), and applying the survey weights produced the same picture. Design-based inference, however, matters for the smaller paths. With MLR and country-clustered standard errors, the standard errors of the pooled paths increased by a factor of 1.4 to 2.7; the associations of digital skills and employer-provided training with wellbeing remained significant (*p* < 0.001), as did the direct association of AI exposure with wellbeing (*p* = 0.001), whereas the three paths to threat appraisal were not significant and the path from threat appraisal to wellbeing was marginal (*p* = 0.07). The delete-one-country jackknife of the WLSMV model, which retains the ordinal estimator, gave the same verdict: jackknife standard errors were 1.9 to 3.3 times the conventional ones; the resource paths (*p* < 0.001) and the AI exposure → wellbeing path (*p* = 0.001) survived; the threat appraisal → wellbeing path was marginal (*p* = 0.045); and the three paths to threat appraisal, including the AI familiarity association, were not significant (*p* ≥ 0.29). The associations on which our conclusions rest are therefore robust to country heterogeneity and clustering; the threat-appraisal paths are not.

For the class contrast, Online Supplementary Materials Table S13 reports the employer-provided training → wellbeing coefficient by class under each specification. A multi-group model with a full set of country fixed effects could not be estimated with the ordinal estimator because several country × class cells contain fewer than ten respondents. Under MLR with country-clustered standard errors, with or without country fixed effects, the ordering was unchanged (working class 0.018–0.020, middle class 0.034–0.037, upper class 0.012–0.014), but the working-versus-middle contrast was not significant (*p* = 0.18–0.19). Under the delete-one-country jackknife, which keeps the ordinal estimator, the contrast remained significant: the working-class coefficient was 0.058 lower than the middle-class coefficient (jackknife SE = 0.023, *p* = 0.012, 95% CI [−0.103, −0.013]); the class-specific R^2^ values kept their ordering (jackknife 95% CIs of [0.04, 0.20], [0.13, 0.28] and [0.17, 0.45] for the working, middle and upper classes); and none of the other six paths differed between the working and middle classes (all *p* ≥ 0.36). The two design-based approaches thus disagree, and the disagreement is informative: MLR treats the four-category training item and the three- and four-category wellbeing indicators as continuous, which attenuates the training coefficient (standardized β = 0.135 versus 0.209 under WLSMV) and, with it, the class difference. We therefore describe the class contrast as consistent in direction and size across estimators, significant under conventional and jackknife inference with the ordinal estimator, and not significant under the continuous indicator clustered specification.

Finally, to check sensitivity to sample composition, we re-estimated the model without the employer training item, which removes the requirement of a valid answer to that item and allows the self-employed, who were not asked it, to enter (Online Supplementary Materials Table S7 and Figure S3). On the same 8065 respondents, the reduced model reproduced every remaining path (digital skills β = 0.271, AI exposure β = −0.056, threat appraisal β = −0.083, all *p* < 0.001), and on the enlarged sample of 9539 respondents, 1347 of them self-employed, the estimates were almost identical (β = 0.273, −0.060 and −0.082). The larger digital skills coefficient in the reduced model reflects the omission of the correlated training item. The main results are therefore not sensitive to the exclusion of the self-employed.

### 4.6. Supplementary Moderation Analysis

H3a and H3b were specified as direct paths from the resources to threat appraisal. Because buffering in the JD-R model refers to an interaction in which resources weaken the effect of demands, we also tested this interaction form with composite scores (item means; wellbeing as the mean of the standardized indicators; all predictors standardized) in regressions estimated with MLR and country-clustered standard errors (Online Supplementary Materials Table S14). With wellbeing as the outcome, the AI exposure × digital skills interaction was positive (b = 0.037, *p* = 0.008): the slope of AI exposure on wellbeing was −0.107 one standard deviation below the mean of digital skills and −0.033 one standard deviation above it. The AI exposure × employer-provided training interaction was close to zero (b = −0.005, *p* = 0.75), and the two interactions added little explained variance (ΔR^2^ = 0.001). With threat appraisal as the outcome, both interactions were positive and small (b = 0.061, *p* = 0.022; b = 0.036, *p* = 0.036) in a model that explained 1% of the variance. Digital skills therefore buffer the association between AI exposure and wellbeing in the interaction sense, whereas employer-provided training is associated with wellbeing directly but does not weaken the AI exposure–wellbeing association. The same composite regressions reproduced the class pattern: the training slope was lower among working-class than among middle-class employees (interaction b = −0.068, *p* = 0.066 with clustered standard errors).

### 4.7. Machine Learning Validation

To test robustness outside parametric assumptions, Random Forest (RF), Regression, and XGBoost using SHAP values were performed. This triangulation of methodology helps confirm that results obtained through the prediction-oriented ML technique will converge with those of the inference-oriented SEM.

**Figure 3:**
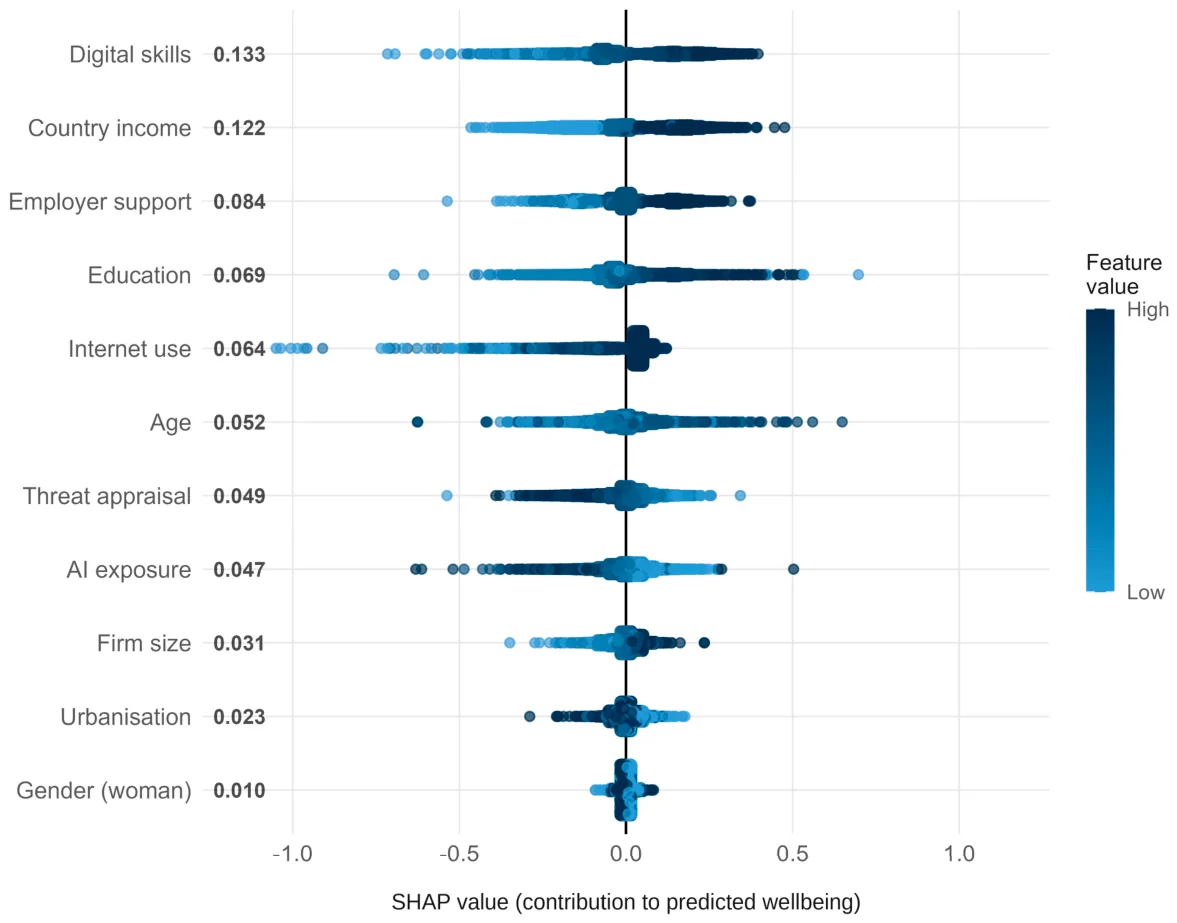
**SHAP summary of the machine learning validation. *Note.*** N = 8065. SHAP values from an XGBoost model (100 rounds; maximum depth, 6; learning rate, 0.1) predicting the wellbeing composite from the four theoretical constructs and seven control variables. Each point is one respondent: its horizontal position is the variable’s contribution to that respondent’s predicted wellbeing, and its color shows the variable’s value (light = low, dark = high). Variables are ordered by mean |SHAP|, printed beside each label (Table S9). Digital skills ranks first (0.133) and employer support (employer-provided digital training) third (0.084), behind country income (0.122). High values of digital skills and employer support (employer-provided digital training) raise predicted wellbeing, whereas high threat appraisal and AI exposure lower it, matching the signs of the structural model.

The ML results converge with the structural model. Random Forest achieved a cross-validated R^2^ of 0.159 (N = 8065), and all four theoretical constructs ranked among the seven most important of the eleven predictors (Online Supplementary Materials Table S8 and Figure S4). Because impurity-based importance understates single-indicator predictors, such as employer-provided digital training, we compare the structural coefficients with mean absolute SHAP values from the XGBoost model (Figure 3 and Online Supplementary Materials Table S9). On this metric, digital skills ranked first (0.133) and employer-provided digital training third (0.084), behind country income (0.122); among the four theoretical constructs, employer-provided digital training ranked second, and threat appraisal (0.049) and AI exposure (0.047) contributed least. The direction of each contribution matched the SEM: higher digital skills and employer-provided digital training raised predicted wellbeing, whereas higher AI exposure and threat appraisal lowered it.

SHAP dependence plots showed largely monotonic relationships for the important variables (See Online Supplementary Materials Figure S6). Employer-provided digital training raised predicted wellbeing most steeply at the upper end of its scale, and threat appraisal lowered predicted wellbeing mainly at high values.

Removing threat appraisal from Random Forest raised the importance of AI exposure by 18.9% (Online Supplementary Materials Table S11), but the importance of most other predictors rose by similar amounts (age by 21.8%), so this comparison does not provide specific evidence of mediation. Class-stratified Random Forests (Online Supplementary Materials Table S10 and Figure S5) reproduced the ordering found in the multi-group model: the cross-validated R^2^ was −0.007 for the working class, 0.152 for the middle class and 0.174 for the upper class, and the importance of digital skills was highest in the upper class.

Congruent results from the parametric and nonparametric analyses indicate that the associations identified are not artifacts of the linear specification of the structural model. In addition, consistency helps mitigate any possible reservations about the linearity specifications within SEM analyses because the ML procedures that impose no functional form constraints obtain substantively similar results.

### 4.8. Hypotheses Summary

Table 5 summarizes the hypothesis tests. Of the nine tests, five hypotheses were supported (H1b, H2a, H2b, H3b, and H5), the class moderation hypothesis was partially supported, and three were not supported. H1a and H4 were not supported because the association between AI exposure and threat appraisal was negative rather than positive, and H3a was not supported because digital skills were unrelated to threat appraisal. The class moderation hypothesis was partially supported: the use of employer-provided digital training to ensure wellbeing differed across classes, with the working class showing the weakest association, whereas the digital skills path and the paths from AI exposure did not differ significantly once the fragile threat-appraisal paths were set aside. The secondary expectation that the middle class would be the most sensitive group was not borne out: the contrast that drives the heterogeneity separates the working class from the middle class.

## 5. Discussion

This research explored the associations between workplace AI, organizational and personal resources, and employee wellbeing using a framework that integrates JD-R theory and Sen’s capability approach. The results support some of the conjectures, contradict others, and reveal a pattern of class heterogeneity with implications for research and workplace practice.

### 5.1. Principal Findings and Their Significance

The findings offer only limited support for treating workplace AI exposure as a job demand in the JD-R sense. Net of resources and the mediating pathway, AI exposure was negatively associated with capability-relevant wellbeing, including financial security and life satisfaction, but the association is small (β = −0.073); the zero-order correlation is close to zero, and AI exposure was not positively related to threat appraisal. The direct association is consistent with the growing literature reporting costs of algorithmic management for workers’ autonomy and wellbeing (Kellogg et al., 2020; Wood et al., 2019; Felix et al., 2023), although studies of collaborative uses of robots and AI report gains in quality of work life and citizenship behavior (Duan et al., 2026a, 2026b), which underlines that the form of deployment matters. Recent evidence from other institutional and labor-market contexts points in the same direction of heterogeneity. Using German panel data for 2000–2020, Giuntella et al. (2025) find no sizeable negative effect of occupational AI exposure on workers’ wellbeing or mental health, and even some improvement in health that they attribute to lower physical job intensity, but a modest and consistent decline in life and job satisfaction among workers who report using AI tools themselves; how workers experience AI appears to matter as much as the tasks it automates. In China, Nie et al. (2026) show that the perceived labor-market consequences of AI exposure, in terms of perceived job replaceability and expected income change, vary with the codifiability of the tasks a worker performs, so that the same exposure carries different implications for different jobs. Read together with our results, these studies suggest that the association between AI exposure and wellbeing is heterogeneous across workers, tasks and labor-market settings rather than a uniform demand effect. Our measure, moreover, records the presence of algorithmic systems rather than the pressure they exert, so the result indicates only that AI exposure can go together with lower wellbeing, not that it operates as a demand in the strain-generating sense. The lesson for organizations is that the implementation of AI is not a neutral technology: it is associated with worker outcomes in measurable, if modest, ways.

The resource hypotheses produced the findings of greatest relevance for workplace practice. Both digital skills and employer-provided digital training were positively associated with wellbeing, as hypothesized, consistent with their status as a personal and an organizational resource, respectively. Employer-provided training had a slightly larger association with wellbeing than digital skills (β = 0.209 versus 0.189). This is consistent with what workplace practitioners have long assumed, namely that developing staff goes together with their wellbeing, although the cross-sectional design does not allow us to say that training produces this effect. The size of the association, moderate in absolute terms but the largest among the workplace variables and robust across all specifications, suggests that training and development are central rather than peripheral to worker wellbeing during technological change.

These results align with the more recent workplace literature that has argued for the importance of developmental practices in creating sources of competitive advantage. There has been evidence of a positive association between investment in training and various outcomes such as productivity, turnover, and worker engagement (Sung & Choi, 2018). In contribution to this line of research, our results suggest that the correlates of training extend beyond traditional organizational outcomes to outcomes that align with Sen’s evaluative framework. Organizational investment in training related to digital technologies and AI can be seen not only as a tool for reducing turnover or increasing engagement, but as associated with the material security and life satisfaction that the capability tradition values. These can be seen to align with systemic reviews that point to worker welfare as an end goal of successful AI-workplace integration (Panda et al., 2025; Úbeda-García et al., 2025).

### 5.2. The AI Familiarity Association

The negative association between AI exposure and threat appraisal is the most surprising result, although we treat it as a secondary finding. It ran contrary to what was expected in the context of the displacement thesis and the popular narrative, as employees who had more exposure to AI experienced lower, not higher, levels of anxiety. The association is small, however (β = −0.037), and the model explains only 0.2% of the variance in threat appraisal; it was also not uniform across classes, being negative among working- and middle-class employees and weakly positive among upper-class employees (Table 4). The association is also not robust to country clustering: under design-based standard errors it is not significant (Section 4.5), so it may partly reflect between-country differences in AI adoption and in attitudes towards AI. The interpretations below should be read with this in mind.

There are a number of interpretations that must be considered. The first, and the most obvious one, is that hands-on experience may demystify AI and reduce threat perception. The popular discourse on AI is apocalyptic, with even expert opinions focusing on massive technological unemployment. People whose only exposure to the topic is through this discourse arguably entertain overstated threat perceptions. Those exposed to the actual usage of the technology experience the limitations as well as the enabling capacities of AI. They realize that AI supplements certain tasks, leaving others the same, that computer predictions need human evaluation, and that their own knowledge is necessary in situations that are ambiguous.

The support for this interpretation comes from studies of uncertainty and anxiety conducted by psychologists. A study by Kim and Lee (2024) showed that while adopting AI heightened job stress, self-efficacy moderated this effect. Perhaps our familiarity association is also a result of this same principle, where practical experience enhances self-efficacy to compensate for job-related anxieties. Similarly, Xu et al. (2023) found that the association between AI awareness and depression runs through emotional exhaustion and is weaker when perceived organizational support is high, which implies that organizations may address job-related anxieties more effectively by providing practical experience and competence than by offering reassurance.

A possible alternative interpretation is based on selection effects. There might be systematic differences between workers in AI-intensive and AI-limited occupations regarding exposure and strain. If workers who are temperamentally adapted to technological change are preferentially allocated AI-related tasks by employers, or if nervous workers self-select out of AI-intensive jobs, the association will represent composition rather than causation. The design is not suited for adjudicating this issue definitively, and we acknowledge this caveat. Given the small size of the association, selection is a plausible explanation that our data cannot rule out.

### 5.3. Direct Versus Indirect Pathways: Implications for Workplace Policy

A crucial aspect of this contribution is the distinction made between direct and indirect effects. The prediction made by the health impairment process within the JD-R model that job demands negatively influence wellbeing due to strain generated, which affects resource depletion and job performance (Bakker & Demerouti, 2017) is not supported by our results in important ways, with the qualification that threat appraisal is a cognitive appraisal rather than a measure of experienced strain (Section 3.2), so that our test concerns the appraisal component of the pathway only.

The indirect effect via threat appraisal turned out to be contrary to expectations, if statistically significant. Since the experience with AI is associated with slightly lower threat appraisal, and threat appraisal is associated with lower wellbeing, the product yields a small positive indirect effect. This positive channel is a limited defense, but it is overpowered by the large negative direct effect, leading to a net negative total effect. AI exposure is associated with lower wellbeing through the direct route rather than through threat appraisal.

What might these direct mechanisms entail? Some possibilities emerge from the literature. AI could limit worker autonomy by issuing direct algorithmic instructions that decrease opportunities for discretionary decision-making roles for workers (Wood et al., 2019). AI could further lower opportunities for skill use by automating tasks where skilled execution occurred before AI adoption. AI-powered worker surveillance could change the subjective experience of work by affecting worker wellbeing in ways not fully accounted for, for example, worker anxiety and producing what Newlands describes as spatial abstraction, and the making invisible of the worker by “datafication” (Newlands, 2021). Algorithmic optimization could facilitate the endless extraction of productivity from the worker by amplifying work demands without corresponding compensation rewards from the organization (Parent-Rocheleau et al., 2024).

The same pattern holds for the resource pathways. Both digital skills and employer-provided digital training are associated with wellbeing almost exclusively through direct paths; the indirect components through threat appraisal are negligible. Digital skills were unrelated to threat appraisal, so their association with wellbeing does not run through lower anxiety. The supplementary moderation analysis (Section 4.6) adds a nuance: digital skills did weaken the negative association between AI exposure and wellbeing, which is the interaction form of buffering, whereas employer-provided training did not. A plausible reading is that skilled workers use AI more effectively rather than feeling less threatened by it, although our data cannot establish this mechanism.

Employer-provided digital training was associated with wellbeing directly and, at most, marginally through lower threat appraisal. One interpretation is that investment in training signals organizational support and meets needs for competence and security independently of anxiety reduction. If so, the implication for organizations is that training should be evaluated primarily on outcomes such as skill use and security rather than on anxiety reduction alone.

The dominance of the direct over the indirect associations could indicate that strategies aimed only at relieving psychological strain may not be sufficient to protect the wellbeing of employees. While alleviating anxiety is certainly a preferred outcome, it does not necessarily eliminate the impact on autonomy and skill depletion that seems to translate into direct negative effects. Studies on platform organization found that worker participation in the implementation of technology is critical in legitimization or coerciveness of algorithmic control (Purcell & Brook, 2022).

Three hypotheses were not supported, and the pattern of failures is informative. H1a and H4 failed because AI exposure was not positively related to threat appraisal, and H3a because digital skills were unrelated to it. Three readings are possible, and they are not mutually exclusive. First, the JD-R health impairment process may not transfer straightforwardly to AI exposure measured as the presence of algorithmic management practices: such exposure may be experienced as a demand by some workers and as routine, or even as a resource, by others, so that its average association with appraisal is close to zero. Second, threat appraisal may be a poor stand-in for strain. Our items record general beliefs about the labor market effects of AI, which are shaped by public discourse and national context (as the sensitivity of these paths to country clustering suggests) more than by personal experience, and which therefore mediate little. Third, the Eurobarometer indicators impose limits: the survey contains no measure of exhaustion, work intensity, or insecurity experienced in one’s own job, which is where the health impairment process would be expected to show. The unsupported hypotheses thus point less to a failure of the JD-R model than to the need for strain measures that are closer to the worker’s own job when the model is applied to AI.

### 5.4. Equal Provision, Unequal Benefit: Class Differences in Conversion

The multi-group results do not support our secondary expectation that the middle class would be the group most sensitive to AI. The direct association between AI exposure and wellbeing was similar across classes. What differed was the use of workplace resources to ensure wellbeing. The same level of employer-provided digital training was associated with a wellbeing difference among working-class employees little more than half as large as among middle-class employees, and the workplace model explained 12.0% of working-class wellbeing compared with 31.1% of upper-class wellbeing. Digital skills showed the same ordering, although not significantly. The working-versus-middle contrast held under country fixed effects and under design-based jackknife standard errors, but it fell short of significance in the continuous indicator specification with country-clustered standard errors (Section 4.5); we therefore regard it as a consistent but not yet conclusive finding. Employers may provide the same training, but the benefit associated with it appears to be unequal; we stress that this is a consistent pattern rather than a statistically conclusive one, and the strength of the claim in the remainder of this section should be read accordingly.

This pattern is what the capability approach would lead one to expect, and conversion factors offer a theoretical explanation for it. They are not a mechanism that our data establish because we do not observe the conversion factors themselves and social class is only a proxy for them. Resources become achieved functionings only through conversion factors, and conversion factors are distributed by social position (Robeyns, 2017). Working-class employees are less likely to hold the complementary assets, such as educational credentials, financial reserves, social connections, and bargaining power, that allow workplace support to be converted into financial security and life satisfaction, and they are more exposed to corrosive disadvantages that spill across domains of life (Wolff & de-Shalit, 2007). The pattern also resembles a Matthew effect of cumulative advantage (Merton, 1968), in which a uniform intervention widens rather than narrows existing gaps because those who already have more are better placed to benefit from it. It is consistent with evidence that technological change operates through class-specific task mechanisms in European labor markets (Gil-Hernández et al., 2024).

For organizations, the implication, if the pattern is confirmed by longitudinal or experimental research, is that equal provision of digital training need not mean equal benefit. Training programs that assume a common baseline of conversion capacity may deliver the smallest returns to the employees with the least support outside work. Rather than redirecting training away from working-class employees, organizations can pair it with complementary supports that strengthen conversion, such as protected time for learning, follow-up coaching and attention to job security during AI implementation, and can evaluate programs by who benefits rather than by participation alone. The lower explained variance for working-class wellbeing is also worth noting, although it should be read cautiously: it shows only that the variables in our model account for less of this group’s wellbeing. Whether the remainder reflects housing security, material constraints, or other non-work conditions, and whether workplace measures alone can therefore close the gap, are questions that the present analysis cannot answer and that future research with richer measures of living conditions should address.

These conclusions rest on the working-versus-middle contrast. The upper-class estimates are less precise, and the upper-class solution contains a Heywood case, so we do not draw conclusions about the upper class on its own.

### 5.5. Limitations and Future Research

There are several limitations to the findings that must be considered. Primarily, the design does not permit causal inference. Although the model is specified with AI exposure and the resources as predictors of wellbeing, reverse causality and unobserved confounding cannot be ruled out. Individuals with lower capabilities might be sorting into environments that make greater use of AI, or something else might be simultaneously affecting the correlation between the two. Longitudinal studies would offer more causal information on the topic.

The use of self-reported data can lead to bias. Common method variance could cause correlations to be inflated. However, results for the measurement model indicate that there is good discriminant validity. Self-reported data could conflate frequency with subjective importance. Similarly, self-reported data for capabilities could reflect adaptive preferences. Future studies could use other methods to understand exposure to AI and other methods for capabilities like income.

A further constraint is the single-item measure of employer-provided digital training, which records whether tools or training are provided but not their quality, intensity, or relevance, and which cannot capture other forms of organizational support such as participation in implementation decisions or job security guarantees. Multi-item measures of AI-related organizational support that make these distinctions are needed.

Furthermore, the European context poses limitations on the generalizability of the study’s findings. This is because the European labor market is known for its stronger labor protections and divergent adoption rates regarding AI, compared with other international regions.

Lastly, for reasons of feasibility, our capability operationalization covers only a fraction of Sen’s capability space. Future studies are advised to work on a measurement tool involving health, socialization, and agency concepts, utilizing frameworks already used in capability measurement studies such as Alkire (2002).

Social class is measured by self-placement, which captures subjective position rather than occupational class, and the upper-class group is small (N = 823), which contributes to the Heywood case in that group. In addition, the main models treat respondents as independent and account for country differences only through the country income control. The robustness checks in Section 4.5 show that country clustering roughly doubles the standard errors, that the paths to threat appraisal do not survive design-based inference, and that the working-versus-middle contrast in the training–wellbeing association survives the jackknife but not the continuous indicator clustered specification. Multi-level models for ordinal indicators with a larger number of countries, or designs with within-country variation in training provision, would provide a stronger test.

## 6. Conclusions

This paper explores the relationship between work-related AI and employee wellbeing. It used a combination of the JD-R Model and Sen’s capability approach. Based on data from over 8000 employees in the EU, this paper examines the association between workplace AI exposure, treated as a potential job demand, and capability-relevant wellbeing. Additionally, the paper examined the role that digital skills and employer-provided digital training play in the relationship, and whether social class conditions it. The results are significant for research and workplace practice.

Three primary findings can be derived from this study. First, workplace AI exposure was weakly and negatively associated with wellbeing, net of resources and controls, but the findings do not establish AI exposure as a job demand in the JD-R sense: the hypothesized strain pathway was not supported (H1a and H4), the model explained almost none of the variance in threat appraisal, and the threat-appraisal paths did not survive country clustering. What the data show is a small direct association between exposure to AI systems, algorithmic allocation, and automated tracking and lower financial security and life satisfaction, an association that is consistent with, but does not demonstrate, mechanisms such as reduced autonomy and skill use, and whose size and direction appear to vary across workers and settings (Giuntella et al., 2025; Nie et al., 2026). For organizations, this means that the wellbeing implications of AI deployment deserve attention, while the mechanisms remain to be established.

Second, and of most relevance for organizations, employer-provided digital training showed the largest association with employee wellbeing among the workplace factors in the model, slightly larger than that of personal digital skills, and this association was robust to every specification examined. The results are consistent with reviews that identify employee development as central to successful AI integration, and they suggest that the training function belongs at the center rather than the margins of technological transformation.

Third, the multi-group analysis suggests that the benefit associated with employer-provided training may be unequally distributed. The association between training and wellbeing among working-class employees was little more than half as large as among middle-class employees, and the workplace model explained less of their wellbeing. This contrast held under country fixed effects and design-based jackknife standard errors but not under the country-clustered specification that treats the indicators as continuous, so it should be regarded as consistent in direction but not statistically conclusive across all specifications. Contrary to our secondary expectation, the middle class was not the most sensitive group; the pattern points to the working class. Therefore, equal provision of employer training may not produce equal benefit, a possibility that warrants confirmation with longitudinal or experimental designs before it is treated as established.

These findings suggest directions for policy that merit further exploration rather than firm prescriptions. Public investment in digital skills and incentives for employer-provided training may be more effective when combined with measures that strengthen conversion among working-class employees than when distributed uniformly on the assumption that equal provision yields equal benefit. The association of employer-provided training with wellbeing points to the potential of instruments that encourage organizational investment in worker development, such as training mandates, tax incentives or co-financing arrangements, although whether such instruments raise wellbeing is a question for evaluation research. At the EU level, initiatives such as the European Skills Agenda and the Digital Decade targets could consider class differences in who benefits from AI-related training when resources are allocated. More broadly, the findings suggest that AI governance frameworks focused solely on job displacement miss a dimension that deserves attention: the wellbeing of workers who remain employed. Regulatory approaches might therefore combine employer obligations for AI-related training with support that helps less advantaged employees benefit from it.

Nonetheless, these results are qualified by several caveats. The cross-sectional nature of the data does not allow causal conclusions to be drawn definitively, and the self-reported variables may to some extent operationalize adaptive preferences instead of objective wellbeing levels. The EU institutional framework might restrict the external validity of the results to other areas with differently shaped labor markets. The upper-class estimates are imprecise, and although the robustness checks address country clustering, the class contrast is not significant under every specification.

In spite of its limitations, this study offers an entry point into the AI–workplace relationship as AI spreads across occupational and sectoral settings. The integration of JD-R theory and Sen’s capability approach offers a framework that articulates mechanisms and accounts for the diversity of AI’s associations with worker outcomes. As companies rapidly expand their use of AI systems, the choices organizations make are likely to matter for whether these systems augment or diminish worker wellbeing. The findings suggest that investment in workers’ digital capabilities goes together with better wellbeing during the adoption of AI, and that such investment is most likely to fulfill its promise when it is designed so that all workers, not only the advantaged, can benefit from it. Whether employer training weakens the costs of AI exposure is a question this design cannot settle: in the supplementary analysis, digital skills, but not employer-provided training, moderated the association between AI exposure and wellbeing.

## Figures and Tables

**Table 1:** Constructs, Measurements, and Reliability.

Construct	Items	Response Scale	α	CR	AVE
Panel A. Latent and observed constructs					
AI exposure	6	1 (never) to 4 (all the time)	0.911	0.934	0.701
Digital skills	4	1 (totally disagree) to 4 (totally agree)	0.897	0.927	0.759
Employer-provided digital training	1	1 (totally disagree) to 4 (totally agree)	—	—	—
Threat appraisal	3	1 (totally disagree) to 4 (totally agree)	0.757	0.804	0.581
Wellbeing	3	1–3/1–4 (mixed)	0.772	0.860	0.686
Panel B. Control variables					
Age	1	Continuous (15–98)	—	—	—
Gender (woman = 1)	1	Binary (0–1)	—	—	—
Education level (ISCED)	1	Ordinal (0–8)	—	—	—
Firm size	1	Ordinal (1–5)	—	—	—
Internet use	1	Continuous (1–6)	—	—	—
Urbanization	1	Ordinal (1–3)	—	—	—
Country income group	1	Categorical (1–3)	—	—	—

***Note***. N = 8115 for the measurement model. α = Cronbach’s alpha; CR = composite reliability; AVE = average variance extracted, both from the confirmatory factor analysis. Employer-provided digital training is a single observed indicator and is therefore excluded from the CFA and entered directly in the structural model; no α, CR, or AVE is estimable for it. One threat-appraisal item (qb6_6_r) was dropped to improve model fit. Full indicator wording, descriptive statistics and control-variable definitions appear in Tables S2 and S5. Source: Special Eurobarometer 101.4.

**Table 2:** Measurement model fit and measurement invariance across social class.

Model/Index	χ^2^	df	CFI	RMSEA	ΔCFI	ΔRMSEA
Panel A. Confirmatory factor analysis						
χ^2^ (df)	1364.44 (98)					
CFI	0.997					
TLI	0.997					
RMSEA [90% CI]	0.040 [0.038, 0.042]					
SRMR	0.036					
Panel B. Measurement invariance across social class						
Configural	1995.11	294	0.9922	0.0463	—	—
Threshold	2281.08	356	0.9911	0.0447	−0.0011	−0.0016
Threshold + loading (metric)	2283.30	380	0.9912	0.0430	0.0001	−0.0017
Threshold + loading + intercept (scalar)	2543.62	404	0.9902	0.0443	−0.0010	0.0013

***Note.*** Panel A: N = 8115; four latent constructs, 16 ordinal indicators, WLSMV estimator. Panel B: N = 8065 (working class = 1454; middle class = 5788; upper class = 823); ordinal invariance sequence following H. Wu and Estabrook (2016). Δ values are relative to the preceding, less restricted model. Invariance is supported at every step by the ΔCFI < 0.010 and ΔRMSEA < 0.015 criteria (F. F. Chen, 2007). Scaled χ^2^ difference tests are significant at each step, as expected at this sample size; ΔCFI is therefore used as the decision rule.

**Table 3:** Structural model results, pooled sample.

Path/Parameter	β	SE	z	*p*
Panel A. Theoretical paths				
AI exposure → threat appraisal	−0.037 **	0.013	−2.81	0.005
Digital skills → threat appraisal	0.004	0.014	0.26	0.799
Employer digital training → threat appraisal	−0.028 *	0.012	−2.28	0.023
AI exposure → wellbeing	−0.073 ***	0.012	−6.07	<0.001
Digital skills → wellbeing	0.189 ***	0.012	15.84	<0.001
Employer digital training → wellbeing	0.209 ***	0.011	19.15	<0.001
Threat appraisal → wellbeing	−0.081 ***	0.012	−6.51	<0.001
Panel B. Control variables predicting wellbeing				
Age	0.022 ^†^	0.012	1.88	0.060
Gender (woman = 1)	−0.026 *	0.012	−2.24	0.025
Education level (ISCED)	0.150 ***	0.012	12.07	<0.001
Firm size	0.054 ***	0.012	4.55	<0.001
Internet use	0.093 ***	0.012	7.60	<0.001
Urbanization	−0.023 *	0.012	−1.99	0.047
Country income group	0.208 ***	0.012	17.93	<0.001
Panel C. Indirect and total effects				
AI exposure → threat appraisal → wellbeing	0.0030 *	0.0012	2.55	0.011
Digital skills → threat appraisal → wellbeing	−0.0003	0.0011	−0.25	0.799
Employer digital training → threat appraisal → wellbeing	0.0023 *	0.0011	2.14	0.032
AI exposure → wellbeing (total)	−0.0695 ***	0.0120	−5.79	<0.001
Digital skills → wellbeing (total)	0.1889 ***	0.0120	15.80	<0.001
Employer digital training → wellbeing (total)	0.2117 ***	0.0110	19.33	<0.001
Panel D. Variance explained				
R^2^ Threat appraisal	0.002			
R^2^ Wellbeing	0.229			

***Note.*** N = 8065. WLSMV estimator with ordinal indicators. Standardized coefficients reported. Model fit: χ^2^(217) = 11,923.55, CFI = 0.967, TLI = 0.982, RMSEA = 0.082 [0.081, 0.083], SRMR = 0.032. Panel D: workplace AI exposure accounts for 0.2% of the variance in threat appraisal, indicating that threat appraisal is essentially independent of direct workplace experience with the technology. Indirect effects are products of unstandardized path coefficients. *** *p* < 0.001, ** *p* < 0.01, * *p* < 0.05, † *p* < 0.10.

**Table 4:** Structural paths by social class and tests of cross-class equality.

Path	Working	Middle	Upper	χ^2^(2)	*p* (Wald)	*p* (Δχ^2^)
AI exposure → threat appraisal	−0.074 *	−0.043 **	0.075 ^†^	9.17	0.010	0.012
Digital skills → threat appraisal	0.060 ^†^	0.013	−0.129 ***	15.17	<0.001	<0.001
Employer digital training → threat appraisal	0.006	−0.031 *	−0.012	1.53	0.465	0.457
AI exposure → wellbeing	−0.023	−0.046 ***	−0.039 ^†^	1.57	0.455	0.428
Digital skills → wellbeing	0.081 ***	0.104 ***	0.142 ***	3.37	0.186	0.192
Employer digital training → wellbeing	0.072 ***	0.130 ***	0.123 ***	11.08	0.004	0.005
Threat appraisal → wellbeing	−0.041 *	−0.036 ***	−0.032	0.12	0.940	0.933
R^2^ wellbeing	0.120	0.203	0.311			
n	1454	5788	823			

***Note*.** N = 8065. Unstandardized coefficients from a multi-group structural equation model (WLSMV) in which all structural paths were freely estimated across classes. χ^2^(2) and *p* (Wald) test the null that a path is equal across all three classes; *p* (Δχ^2^) is the corresponding scaled χ^2^ difference test. Omnibus test that all seven paths are equal across classes: Δχ^2^(14) = 33.36, *p* = 0.003. For employer digital training → wellbeing, the significant pairwise contrast is middle versus working class, *p* = 0.0009. Upper-class estimates should be interpreted with caution: the solution for that group contains a Heywood case (negative residual variance on one wellbeing indicator) arising from sparse response categories in the smallest group. *** *p* < 0.001, ** *p* < 0.01, * *p* < 0.05, † *p* < 0.10.

**Table 5:** Summary of hypothesis tests.

H	Path	Expected	Estimate	*p*	Outcome
H1a	AI exposure → threat appraisal	+	−0.037	0.005	Not supported (opposite sign)
H1b	AI exposure → wellbeing (direct)	-	−0.073	<0.001	Supported
H2a	Digital skills → wellbeing	+	0.189	<0.001	Supported
H2b	Employer digital training → wellbeing	+	0.209	<0.001	Supported
H3a	Digital skills → threat appraisal	-	0.004	0.799	Not supported
H3b	Employer digital training → threat appraisal	-	−0.028	0.023	Supported
H4	AI exposure → threat appraisal → wellbeing	-	Indirect = +0.003	0.011	Not supported (opposite sign)
H5	Threat appraisal → wellbeing	-	−0.081	<0.001	Supported
CM	Cross-class equality of structural paths	Heterogeneous	χ^2^(2) = 11.08	0.004	Partially supported

***Note.*** N = 8065. Estimates are standardized coefficients from the pooled structural model (Table 3), except for the final row, which reports the cross-class equality test for employer digital training → wellbeing from Table 4. H1a predicted that AI exposure would raise threat appraisal; the observed association is negative, and the model accounts for 0.2% of the variance in threat appraisal, so the displacement premise is not supported. H4 predicted a negative indirect effect; the observed indirect effect is positive because the exposure–appraisal association is negative. CM = class moderation.

## Data Availability

The data are available from GESIS at https://search.gesis.org/research_data/ZA8844 (https://doi.org/10.4232/1.14471) (accessed on 25 June 2025). The R code used for the analyses is available from the corresponding author on request.

## Supplementary Materials

The following supporting information can be downloaded at https://www.mdpi.com/article/10.3390/bs16101899/s1. Table S1. Sample construction and social class distribution. Table S1b. Retained versus excluded respondents among employed respondents with complete indicator data. Table S2. Indicator wording, response scales and descriptive statistics (structural-model sample, n = 8065). Table S3. Standardised factor loadings from the confirmatory factor analysis. Table S4. Latent factor correlations and discriminant validity. Table S5. Control variable definitions and measurement. Table S6. Indirect and total effects by social class. Table S7. Robustness check: reduced model without employer digital training, same and enlarged samples. Table S8. Random forest variable importance, full ranking. Table S9. Convergence between the structural model and the machine-learning validation. Table S10. Class-stratified random forest results. Table S11. Random forest with and without threat appraisal. Table S12. Pooled structural paths under alternative treatments of country heterogeneity and survey design. Table S13. Employer digital training → wellbeing by social class under conventional and design-based inference. Table S14. Supplementary moderation analysis with composite scores (MLR, country-clustered standard errors). Figure S1. Structural model with standardised coefficients. Figure S2. Structural paths by social class. Figure S3. Robustness check: main model versus reduced model without employer digital training. Figure S4. Random forest variable importance. Figure S5. Random forest variable importance by social class. Figure S6. SHAP dependence plots for the four theoretical constructs.
