# Supplementary material for: Equal Provision, Unequal Benefit: Social Class and the Conversion of Employer-Provided Digital Training into Worker Wellbeing Across 27 European Countries
Source: Behav Sci (Basel). 2026 Oct 14;16(10):1899. doi: 10.3390/bs16101899 (PMC13701285; doi:10.3390/bs16101899)
Supplement: Supplementary file 1 [file behavsci-16-01899-s001.zip › behavsci-4606455-supplementary.pdf]

Equal Provision, Unequal Benefit: Social Class and the Conversion of Employer Provided Digital Training into Worker Wellbeing Across 27 European Countries

Appendix

Supplementary Tables (Tables S1, S1b, S2–S14) and Figures (Figures S1–S6)

Table S1.

Sample construction and social class distribution

Step	n	Excluded	Reason for exclusion
1. Full Eurobarometer 101.4 sample	26,404	—	
2. Currently employed (d15a_r2 = 1–4)	13,883	12,521	Not currently employed (house persons, unemployed, retired, students)
3. Complete on the 16 latent-construct indicators	9,664	4,219	Missing, "not applicable" or "don't know" on AI exposure, digital skills, threat appraisal or wellbeing indicators
4a. Employees (work for an employer)	8,285	1,379	Self-employed: the employer digital training item (QB3) is asked only of respondents who work for an employer
4b. Valid answer on the employer digital training item	8,153	132	"Not applicable" (93) or "don't know" (39) on QB3
5. Valid self-placed social class (measurement-model sample)	8,115	38	Other, none, refusal or don't know on social class (D63)
6. Complete on the seven control variables (structural-model sample)	8,065	50	Firm size (31), gender (16), education (2), internet use (1)
Social class distribution, structural-model sample			
Working class	1,454	18.0%	
Middle class	5,788	71.8%	
Upper class	823	10.2%	

Note. The measurement model (Table 2, Panel A) is estimated on n = 8,115; all structural models (Tables 3 and 4) on n = 8,065 after listwise deletion on the control variables. Steps 4a and 4b account for most of the reduction between steps 3 and 5: the employer digital training item is asked only of employees, so the analytical sample is a sample of employees rather than of all working people. Table S1b compares retained and excluded respondents; Table S7 replicates the model on the enlarged sample that includes the self-employed. Source: Special Eurobarometer 101.4.

Table S1b.

Retained versus excluded respondents among employed respondents with complete indicator data

Variable	Retained (n = 8,065) M (SD)	Excluded (n = 1,599) M (SD)	Difference	Cohen's d	p	Excluded self-employed (n = 1,379) M	Excluded employees (n = 220) M
Age (years)	43.79 (11.71)	47.12 (12.12)	−3.33	−0.28	< .001	47.32	45.88
Woman (share)	0.53 (0.50)	0.40 (0.49)	0.13	0.25	< .001	0.38	0.53

Variable	Retained (n = 8,065) M (SD)	Excluded (n = 1,599) M (SD)	Difference	Cohen's d	p	Excluded self-employed (n = 1,379) M	Excluded employees (n = 220) M
Education (ISCED 0–8)	4.39 (1.79)	4.53 (1.84)	−0.14	−0.08	.005	4.58	4.19
Firm size 50+ persons (share)	0.34 (0.47)	0.08 (0.26)	0.27	0.60	< .001	0.06	0.18
Internet use (1–6)	5.68 (0.77)	5.66 (0.85)	0.01	0.02	.548	5.71	5.36
Large town (share)	0.34 (0.47)	0.33 (0.47)	0.01	0.03	.358	0.33	0.31
Lower-income EU country (share)	0.31 (0.46)	0.31 (0.46)	0.00	0.00	.902	0.30	0.34
Higher-income EU country (share)	0.36 (0.48)	0.28 (0.45)	0.08	0.16	< .001	0.28	0.32
Working class (share)	0.18 (0.38)	0.15 (0.36)	0.03	0.08	.006	0.14	0.23
Upper class (share)	0.10 (0.30)	0.14 (0.35)	−0.04	−0.14	< .001	0.15	0.07
AI exposure (1–4, item mean)	2.02 (0.83)	1.75 (0.84)	0.27	0.32	< .001	1.77	1.64
Digital skills (1–4, item mean)	3.03 (0.72)	2.95 (0.78)	0.08	0.11	< .001	2.97	2.83
Threat appraisal (1–4, item mean)	2.84 (0.72)	2.90 (0.74)	−0.06	−0.08	.003	2.87	3.10
Wellbeing (mean of z-scored indicators)	0.01 (0.80)	−0.03 (0.88)	0.04	0.05	.112	0.00	−0.23

Note. Base: the 9,664 employed respondents with complete data on the 16 indicators (step 3 of Table S1). Retained = structural-model sample; excluded = respondents lost at steps 4a to 6, of whom 1,379 are self-employed and 220 are employees with a missing answer on the training item, social class or a control variable. Cohen's d is the standardised mean difference (retained minus excluded); p from Welch t tests for continuous variables and two-sample tests of proportions for shares. Composites are item means; wellbeing is the mean of the z-scored indicators. Class shares are computed among respondents with a valid social class. The excluded group differs from the retained sample mainly in ways that follow from self-employment (age, gender, firm size, AI exposure); it does not differ in wellbeing.

Table S2.

Indicator wording, response scales and descriptive statistics (structural-model sample, n = 8,065)

Indicator	Item wording (Special Eurobarometer 101.4)	Response scale (coded)	M	SD	Skew	Kurtosis
AI exposure (QB7)	"Have the following activities ever been performed by digital technologies, including Artificial Intelligence, in your current or previous workplaces? For each activity, please refer to your most recent workplace experience."	1 = no, never; 2 = no, rarely; 3 = yes, often; 4 = yes, all the time				
qb7_1_r	Hiring workers	as above	1.84	0.96	0.74	−0.71
qb7_2_r	Allocating tasks to workers	as above	1.96	1.00	0.54	−1.01

Indicator	Item wording (Special Eurobarometer 101.4)	Response scale (coded)	M	SD	Ske w	Kur tosis
qb7_3_r	Managing worktime schedules	as above	2.1 4	1.0 5	0.2 9	−1.2 6
qb7_4_r	Monitoring workers' activities	as above	2.0 8	1.0 3	0.4 0	−1.1 4
qb7_5_r	Assessing workers' performance, including imposing sanctions or attributing rewards	as above	1.9 4	1.0 0	0.6 1	−0.9 0
qb7_6_r	Enforcing safety measures	as above	2.1 7	1.0 6	0.2 7	−1.2 5
Digital skills (QB2)	"To what extent do you agree or disagree with the following statements regarding your skills in the use of the most recent digital technologies, including Artificial Intelligence? … You consider yourself to be sufficiently skilled in the use of digital technologies …"	1 = totally disagree; 2 = tend to disagree; 3 = tend to agree; 4 = totally agree				
qb2_1_r	… in your daily life	as above	3.0 4	0.8 3	−0. 68	0.04
qb2_2_r	… to do your job	as above	3.0 4	0.8 4	−0. 70	0.00
qb2_3_r	… if you were to find a job or to change jobs within the next twelve months	as above	2.9 9	0.8 4	−0. 60	−0.1 3
qb2_4_r	… to benefit from digital and online learning opportunities	as above	3.0 3	0.8 3	−0. 65	−0.0 3
Employer digital training (QB3)	"To what extent do you agree or disagree that your employer provides you with the necessary tools or training to work effectively with the most recent digital technologies, including Artificial Intelligence?" (respondents with a current occupation who work for an employer)	1 = totally disagree to 4 = totally agree				
qb3_r	(single item)	as above	2.9 4	0.8 3	−0. 56	−0.1 2
Threat appraisal (QB6)	"To what extent do you agree or disagree with each of the following statements?"	1 = totally disagree to 4 = totally agree				
qb6_1_r	Due to the use of robots and artificial intelligence, more jobs will disappear than new jobs will be created	as above	2.9 1	0.8 7	−0. 37	−0.6 3
qb6_5_r	Robots and Artificial Intelligence steal people's jobs	as above	2.8 3	0.9 0	−0. 33	−0.6 8
qb6_7_r	Robots and Artificial Intelligence have a negative impact on communication between colleagues	as above	2.7 7	0.8 9	−0. 25	−0.7 2
Wellbeing						
d60_r	During the last twelve months, would you say you had difficulties to pay your bills at the end of the month? (D60)	1 = most of the time; 2 = from time to	2.6 2	0.5 8	−1. 22	0.47

Indicator	Item wording (Special Eurobarometer 101.4)	Response scale (coded)	M	SD	Skew	Kurtosis
		time; 3 = almost never or never				
d70_r	On the whole, are you very satisfied, fairly satisfied, not very satisfied or not at all satisfied with the life you lead? (D70)	1 = not at all satisfied; 2 = not very satisfied; 3 = fairly satisfied; 4 = very satisfied	3.15	0.61	−0.35	0.73
d70a_r	Satisfaction with daily life, same response format (D70a)	as above	3.14	0.61	−0.33	0.65
Social class (D63)	"Do you see yourself and your household belonging to …?"	Working class (1); lower middle and middle class (2); upper middle and higher class (3)				

Note. n = 8,065 (structural-model sample). Wording from the Special Eurobarometer 554 questionnaire (European Commission, 2025); the QB2 and QB6 stems are abbreviated. All items were coded so that higher values indicate more of the construct (QB2, QB3, QB6, D70 and D70a were reverse coded); spontaneous "not applicable", "don't know" and "refusal" answers were treated as missing. A fourth threat-appraisal item (QB6_6, "Robots and Artificial Intelligence increase the pace at which workers complete tasks") was dropped to improve model fit. Descriptive statistics for the measurement-model sample (n = 8,115) are practically identical and are available from the authors.

Table S3.

Standardised factor loadings from the confirmatory factor analysis

Construct	Indicator	Standardised loading	SE	z	p
AI exposure	qb7_1_r	0.822	0.005	179.42	< .001
AI exposure	qb7_2_r	0.875	0.004	247.56	< .001
AI exposure	qb7_3_r	0.834	0.004	196.06	< .001
AI exposure	qb7_4_r	0.841	0.004	201.09	< .001
AI exposure	qb7_5_r	0.852	0.004	214.51	< .001
AI exposure	qb7_6_r	0.799	0.005	163.05	< .001
Digital skills	qb2_1_r	0.876	0.003	251.23	< .001
Digital skills	qb2_2_r	0.880	0.003	262.62	< .001
Digital skills	qb2_3_r	0.854	0.004	221.66	< .001
Digital skills	qb2_4_r	0.875	0.003	252.04	< .001
Threat appraisal	qb6_1_r	0.776	0.008	98.94	< .001
Threat appraisal	qb6_5_r	0.858	0.007	116.14	< .001
Threat appraisal	qb6_7_r	0.636	0.008	78.62	< .001
Wellbeing	d60_r	0.514	0.012	42.68	< .001
Wellbeing	d70_r	0.976	0.007	136.29	< .001

Wellbeing	d70a_r	0.917	0.007	129.38	< .001

Note. N = 8,115. WLSMV estimator. The loading of d70_r on wellbeing (.976) approaches unity; this indicator is also the source of the Heywood case in the upper-class group reported in Table 4.

Table S4.

Latent factor correlations and discriminant validity

Construct pair / construct	r / AVE	SE / √AVE	p
Panel A. Latent factor correlations			
AI exposure – Digital skills	0.185	0.012	< .001
AI exposure – Threat appraisal	-0.049	0.013	< .001
AI exposure – Wellbeing	0.008	0.013	.550
Digital skills – Threat appraisal	-0.082	0.013	< .001
Digital skills – Wellbeing	0.381	0.011	< .001
Threat appraisal – Wellbeing	-0.144	0.013	< .001
Panel B. Fornell–Larcker criterion			
AI exposure	0.701	0.837	
Digital skills	0.759	0.871	
Threat appraisal	0.581	0.762	
Wellbeing	0.686	0.828	

Note. N = 8,115. Panel B: the square root of the average variance extracted exceeds every corresponding inter-factor correlation in Panel A, supporting discriminant validity.

Table S5.

Control variable definitions and measurement

Variable	Definition	Measurement
Age	Respondent age in years. Standard demographic control.	Continuous (15-98)
Gender	Self-reported gender identity. Gender inequality control.	Binary: 0=Man (reference), 1=Woman
Education Level	Highest completed education level using ISCED classification. Fundamental human capital measure.	Ordinal (0-8): 0=No education to 8=Doctorate
Firm Size	Number of employees at local workplace site. Organizational resource capacity indicator.	Ordinal (1-5): 1=Solo to 5=250+ persons
Internet Use	Average frequency of internet use across three contexts: home, work, and mobile. Digital engagement indicator.	Continuous index (1-6): 1=Never to 6=Everyday
Urbanization	Settlement type measuring urban-rural gradient. Infrastructure and opportunity access proxy.	Ordinal (1-3): 1=Rural to 3=Large town
Country Income Group	Countries grouped by GDP per capita PPP into terciles. Alternative to country fixed effects.	Ordinal (1-3): 1=Lower to 3=Higher income EU

Note. Internet use is a composite index constructed from three Eurobarometer items (d62_1, d62_2, d62_3) measuring internet use frequency at home, at work, and on mobile devices. The original response scale (1 = every day to 6 = never, 7 = no access) was reverse coded so that higher values indicate more frequent use, and "no access" responses were coded as missing; the index is the average across the three contexts. Country income groupings are based on GDP per capita (PPP): lower-income EU includes Poland, Hungary, Latvia, Croatia, Greece, Romania and Bulgaria; middle-income EU includes Malta, Italy, Spain, Cyprus, Czechia, Slovenia, Estonia, Portugal, Lithuania and Slovakia; higher-income EU includes Luxembourg, Ireland, the Netherlands, Denmark, Austria, Sweden, Germany, Belgium, Finland and France. Source: Special Eurobarometer 101.4.

Table S6.

Indirect and total effects by social class

Effect	Estimate	SE	p	95% CI
Panel A. Indirect effects on wellbeing				
AI exposure — Working class	0.00306†	0.00180	.090	[-0.00047, 0.00659]
AI exposure — Middle class	0.00156*	0.00067	.020	[0.00025, 0.00287]
AI exposure — Upper class	-0.00240	0.00193	.214	[-0.00619, 0.00138]
Digital skills — Working class	-0.00247	0.00165	.133	[-0.00570, 0.00075]
Digital skills — Middle class	-0.00048	0.00057	.401	[-0.00159, 0.00063]
Digital skills — Upper class	0.00415	0.00289	.151	[-0.00152, 0.00981]
Employer digital training — Working class	-0.00024	0.00111	.828	[-0.00242, 0.00194]
Employer digital training — Middle class	0.00111†	0.00058	.055	[-0.00002, 0.00224]
Employer digital training — Upper class	0.00039	0.00118	.741	[-0.00192, 0.00270]
Panel B. Total effects on wellbeing				
AI exposure — Working class	-0.02037	0.01627	.211	[-0.05226, 0.01152]
AI exposure — Middle class	-0.04475***	0.00848	< .001	[-0.06137, -0.02814]
AI exposure — Upper class	-0.04137*	0.02062	.045	[-0.08180, -0.00095]
Digital skills — Working class	0.07840***	0.01740	< .001	[0.04430, 0.11251]
Digital skills — Middle class	0.10384***	0.00891	< .001	[0.08638, 0.12131]
Digital skills — Upper class	0.14640***	0.03036	< .001	[0.08689, 0.20591]
Employer digital training — Working class	0.07194***	0.01494	< .001	[0.04266, 0.10121]
Employer digital training — Middle class	0.13107***	0.00904	< .001	[0.11336, 0.14879]
Employer digital training — Upper class	0.12327***	0.02657	< .001	[0.07120, 0.17534]

Note. N = 8,065. Unstandardised effects from the multi-group model with all structural paths freely estimated. Indirect effects run through threat appraisal. Classes are ordered working, middle, upper.

Table S7.

Robustness check: reduced model without employer digital training, same and enlarged samples

Path / index	Main model, n = 8,065 β (p)	Reduced model, same sample, n = 8,065 β (p)	Reduced model, enlarged sample, n = 9,539 β (p)
AI exposure → threat appraisal	−0.037 (.005)	−0.039 (.003)	−0.058 (< .001)
Digital skills → threat appraisal	0.004 (.799)	−0.005 (.708)	−0.019 (.133)
Employer digital training → threat appraisal	−0.028 (.023)	—	—
AI exposure → wellbeing	−0.073 (< .001)	−0.056 (< .001)	−0.060 (< .001)
Digital skills → wellbeing	0.189 (< .001)	0.271 (< .001)	0.273 (< .001)
Employer digital training → wellbeing	0.209 (< .001)	—	—
Threat appraisal → wellbeing	−0.081 (< .001)	−0.083 (< .001)	−0.082 (< .001)
R^2 wellbeing	0.229	0.221	0.227
CFI	0.967	0.989	0.988
TLI	0.982	0.993	0.993
RMSEA	0.082	0.053	0.056
SRMR	0.032	0.033	0.033

Note. Standardised coefficients (p in parentheses), WLSMV estimator with ordinal indicators and the seven control variables. The reduced model omits the employer digital training item, which removes the requirement of a valid answer to that item and allows the self-employed (n = 1,347), who were not asked it, to enter the enlarged sample. All sample sizes are after listwise deletion on the control variables. Every path retains its sign and significance; the larger digital skills coefficient in the reduced model reflects the omission of the correlated training item.

Table S8.

Random forest variable importance, full ranking

Rank	Variable	Importance
1	Age	0.1813
2	AI exposure	0.1332
3	Digital skills	0.1309
4	Threat appraisal	0.1156
5	Education	0.0914
6	Firm size	0.0694
7	Employer digital training	0.0682
8	Country income	0.0611
9	Internet use	0.0607
10	Urbanisation	0.0550
11	Gender (woman)	0.0332

Note. n = 8,065; 500 trees; cross-validated $R^2 = 0.159$. Importance is the mean decrease in node impurity. This metric is not a partial effect and systematically under-weights single-indicator predictors relative to multi-item latent constructs; see Table S9.

Table S9.

Convergence between the structural model and the machine-learning validation

Variable	SEM β	Direction	Mean |SHAP|	SHAP rank	RF importance	RF rank
AI exposure	-0.073	Negative	0.047	8	0.133	2
Digital skills	0.189	Positive	0.133	1	0.131	3
Employer digital training	0.209	Positive	0.084	3	0.068	7
Threat appraisal	-0.081	Negative	0.049	7	0.116	4
Education	0.150	Positive	0.069	4	0.091	5
Country income	0.208	Positive	0.122	2	0.061	8

Note. n = 8,065. SEM β are standardised coefficients from the pooled structural model (Table 3). Mean |SHAP| is from an XGBoost model (100 rounds, maximum depth 6, learning rate 0.1) predicting the wellbeing composite; ranks are out of all 11 predictors. Mean |SHAP| is the appropriate comparison metric because, like the structural coefficient, it measures a variable's contribution to individual predictions. On that metric digital skills ranks first and employer digital training third, behind country income; among the four theoretical constructs employer digital training ranks second. Threat appraisal and AI exposure contribute least of the four, consistent with their small structural coefficients. Random forest impurity importance is reported for completeness but ranks employer digital training lower, because employer digital training enters as a single ordinal indicator whereas the remaining theoretical constructs are composites of three to six items.

Table S10.

Class-stratified random forest results

Variable / class	Working	Middle	Upper
Cross-validated R^2	-0.007	0.152	0.174
Variable importance by class			

Age	0.179	0.180	0.158
AI exposure	0.125	0.135	0.132
Digital skills	0.125	0.130	0.170
Threat appraisal	0.118	0.115	0.123
Education	0.090	0.085	0.092
Employer digital training	0.060	0.073	0.069
Firm size	0.072	0.071	0.063
Country income	0.054	0.063	0.075
Internet use	0.087	0.058	0.038
Urbanisation	0.056	0.057	0.052
Gender (woman)	0.034	0.033	0.028

Note. Group sizes: working class = 1,454; middle class = 5,788; upper class = 823. Separate random forests (500 trees) were fitted within each class; importance is the mean decrease in node impurity. A negative cross-validated R^2 means out-of-sample prediction is worse than the class mean. Digital skills importance and model R^2 both rise with class position, consistent with the class pattern in Table 4 and Figure 2.

Table S11.

Random forest with and without threat appraisal

	With threat appraisal	Without threat appraisal	% change
Cross-validated R^2	0.159	0.144	
Variable importance			
Age	0.181	0.221	21.8%
AI exposure	0.133	0.158	18.9%
Digital skills	0.131	0.143	9.5%
Education	0.091	0.101	10.5%
Firm size	0.069	0.078	12.1%
Urbanisation	0.055	0.062	12.8%
Gender (woman)	0.033	0.037	12.8%
Internet use	0.061	0.064	5.9%
Country income	0.061	0.064	4.8%
Employer digital training	0.068	0.071	3.9%

Note. n = 8,065. Removing threat appraisal lowers the cross-validated R^2 by .015 and raises the importance of every remaining predictor. The increase for AI exposure (18.9%) is not distinctive: age rises by 21.8%. The comparison therefore does not provide specific evidence of mediation through threat appraisal; it mainly reflects the redistribution of impurity importance when a correlated predictor is removed.

Table S12.

Pooled structural paths under alternative treatments of country heterogeneity and survey design

**Path**	**Main: WLSMV β (p)**	**WLSMV + country FE β (p)**	**WLSMV weighted β (p)**	**MLR clustered β (p)**	**MLR clustered + country FE β (p)**	**WLSMV, jackknife SE β (p)**
AI exposure → threat appraisal	−0.037 (.005)	−0.055 (< .001)	−0.028 (.051)	−0.021 (.523)	−0.021 (.523)	−0.037 (.290)
Digital skills → threat appraisal	0.004 (.799)	0.013 (.363)	−0.022 (.130)	−0.048 (.305)	−0.048 (.305)	0.004 (.923)

Path	Main: WLSMV β (p)	WLSMV + country FE β (p)	WLSMV weighted β (p)	MLR clustered β (p)	MLR clustered + country FE β (p)	WLSMV, jackknife SE β (p)
Employer digital training → threat appraisal	−0.028 (.023)	−0.034 (.008)	−0.042 (.002)	−0.035 (.111)	−0.035 (.111)	−0.028 (.358)
AI exposure → wellbeing	−0.073 (< .001)	−0.046 (< .001)	−0.078 (< .001)	−0.058 (.001)	−0.034 (.034)	−0.073 (.001)
Digital skills → wellbeing	0.189 (< .001)	0.153 (< .001)	0.190 (< .001)	0.199 (< .001)	0.164 (< .001)	0.189 (< .001)
Employer digital training → wellbeing	0.209 (< .001)	0.181 (< .001)	0.205 (< .001)	0.135 (< .001)	0.122 (< .001)	0.209 (< .001)
Threat appraisal → wellbeing	−0.081 (< .001)	−0.054 (< .001)	−0.082 (< .001)	−0.066 (.071)	−0.041 (.148)	−0.081 (.045)

Note. n = 8,065. Standardised coefficients with p values in parentheses. Country FE: 27 country-sample dummies (reference: Germany West) replace the country income group control; East and West Germany are separate Eurobarometer samples. Weighted: Eurobarometer post-stratification weight (w1). MLR clustered: robust maximum likelihood treating the indicators as continuous, with standard errors clustered by country sample (28 clusters); the country-clustered standard errors are 1.4 to 2.7 times the unclustered MLR standard errors. Jackknife: WLSMV point estimates with standard errors from a delete-one-country jackknife (28 refits, each omitting one country sample); jackknife standard errors are 1.9 to 3.3 times the conventional WLSMV standard errors. Fit of the WLSMV + country FE model: CFI = 0.954, TLI = 0.991, RMSEA = 0.054, SRMR = 0.027; R^2 wellbeing = 0.272.

Table S13.

Employer digital training → wellbeing by social class under conventional and design-based inference

Specification	Working b (SE)	Middle b (SE)	Upper b (SE)	Omnibus p	p W vs M	p M vs U	p W vs U
Main model: WLSMV, conventional SEs	0.072 (0.015)	0.130 (0.009)	0.123 (0.026)	.004	.001	.800	.096
MLR, country-clustered SEs	0.020 (0.010)	0.037 (0.008)	0.014 (0.007)	.101	.188	.036	.643
MLR + country fixed effects, country-clustered SEs	0.018 (0.009)	0.034 (0.007)	0.012 (0.006)	.068	.182	.022	.585
WLSMV, delete-one-country jackknife SEs	0.072 (0.021)	0.130 (0.019)	0.123 (0.039)	—	.012	.864	.215

Note. Unstandardised coefficients (standard errors) for employer digital training → wellbeing from multi-group models with all structural paths freely estimated (working class n = 1,454; middle class n = 5,788; upper class n = 823). Omnibus p: Wald test of equality across the three classes (2 df). Pairwise p values are Wald tests (first three rows) or z tests on the jackknife standard error of the difference (last row; working minus middle = −0.058, SE = 0.023, 95% CI [−0.103, −0.013]). A multi-group WLSMV model with country fixed effects did not converge, because several country × class cells contain fewer than ten respondents. MLR coefficients are on the continuous-indicator scale and are not comparable in size with the WLSMV coefficients. Under the jackknife, no other structural path differed between the working and middle classes (all p ≥ .36), and the R^2 for wellbeing was 0.120 [0.041, 0.199], 0.203 [0.127, 0.278] and 0.311 [0.175, 0.447] for the working, middle and upper classes (95% jackknife CIs).

Table S14.

Supplementary moderation analysis with composite scores (MLR, country-clustered standard errors)

Model	Predictor	b	SE	p
M1 Wellbeing: main effects (R^2 = .177)	AI exposure	−0.066	0.019	< .001
	Digital skills	0.166	0.027	< .001

Model	Predictor	b	SE	p
	Employer digital training	0.125	0.021	< .001
	Threat appraisal	−0.066	0.029	.025
M2 Wellbeing: + AI exposure × resources (R^2 = .178)	AI exposure	−0.070	0.020	< .001
	Digital skills	0.173	0.028	< .001
	Employer digital training	0.124	0.021	< .001
	Threat appraisal	−0.069	0.028	.015
	AI exposure × digital skills	0.037	0.014	.008
	AI exposure × employer digital training	−0.005	0.016	.749
	Joint Wald test of the two interactions	$\chi^2(2)$ = 7.50		.024
M3 Threat appraisal: main effects (R^2 = .005)	AI exposure	−0.015	0.027	.588
	Digital skills	−0.048	0.038	.208
	Employer digital training	−0.030	0.019	.125
M4 Threat appraisal: + AI exposure × resources (R^2 = .012)	AI exposure	−0.027	0.025	.277
	Digital skills	−0.037	0.037	.311
	Employer digital training	−0.020	0.018	.270
	AI exposure × digital skills	0.061	0.027	.022
	AI exposure × employer digital training	0.036	0.017	.036
	Joint Wald test of the two interactions	$\chi^2(2)$ = 11.58		.003
M5 Wellbeing: resources × class, middle class = reference (R^2 = .193)	Employer digital training	0.141	0.025	< .001
	Employer digital training × working class	−0.068	0.037	.066
	Employer digital training × upper class	−0.052	0.042	.209
	Digital skills	0.157	0.030	< .001
	Digital skills × working class	−0.023	0.038	.546
	Digital skills × upper class	0.106	0.059	.073
	AI exposure	−0.069	0.022	.002
	AI exposure × working class	0.007	0.050	.896
	AI exposure × upper class	0.006	0.037	.876
	Working class	−0.133	0.045	.004
	Upper class	0.347	0.057	< .001
	Joint Wald test: training × class	$\chi^2(2)$ = 3.79		.150

Note. n = 8,065. Composite scores are item means (wellbeing: mean of the z-scored indicators); all predictors were standardised before the products were formed, so b is the change in the outcome (in SD units) per SD of the predictor. All models include the seven control variables (not shown). Estimator: robust maximum likelihood with standard errors clustered by country sample (28

clusters). Simple slopes of AI exposure on wellbeing in M2: −0.107 at one SD below the mean of digital skills, −0.070 at the mean and −0.033 at one SD above; −0.065, −0.070 and −0.075 at the corresponding levels of employer digital training.

Figure S1.

Structural model with standardised coefficients

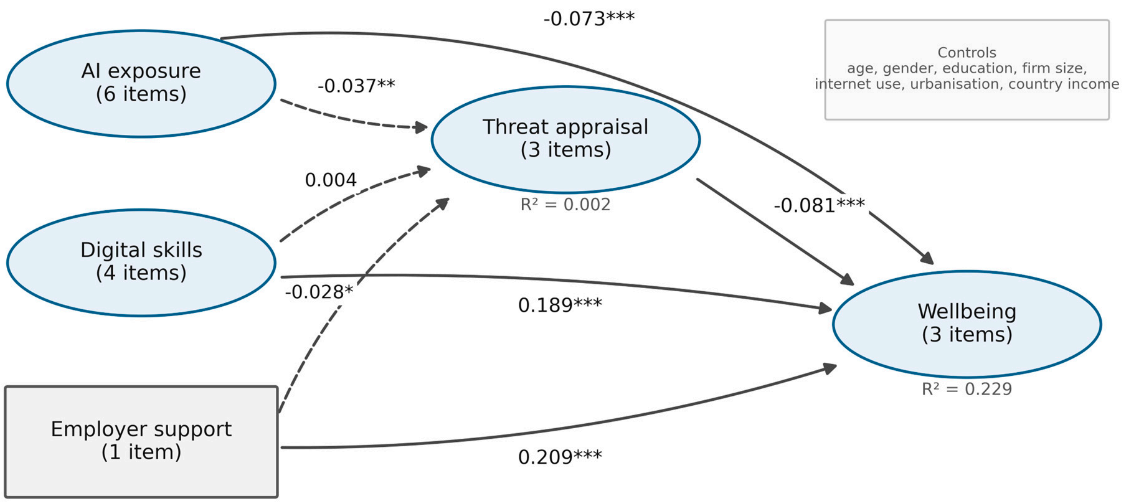

Note. N = 8,065. Standardised coefficients from the pooled structural equation model (WLSMV estimator, ordinal indicators). Solid arrows denote paths to wellbeing; dashed arrows denote paths to threat appraisal. Ellipses are latent constructs and the rectangle is a single observed indicator. Control variables predict wellbeing only and are summarised in the box rather than drawn. Model fit: $\chi^2(217) = 11923.55$, CFI = .967, TLI = .982, RMSEA = .082 [.081, .083], SRMR = .032. The R^2 of .002 for threat appraisal indicates that workplace AI exposure, digital skills and employer digital training jointly account for almost none of the variance in how threatening workers find AI. *** $p < .001$, ** $p < .01$, * $p < .05$.

Figure S2.

Structural paths by social class

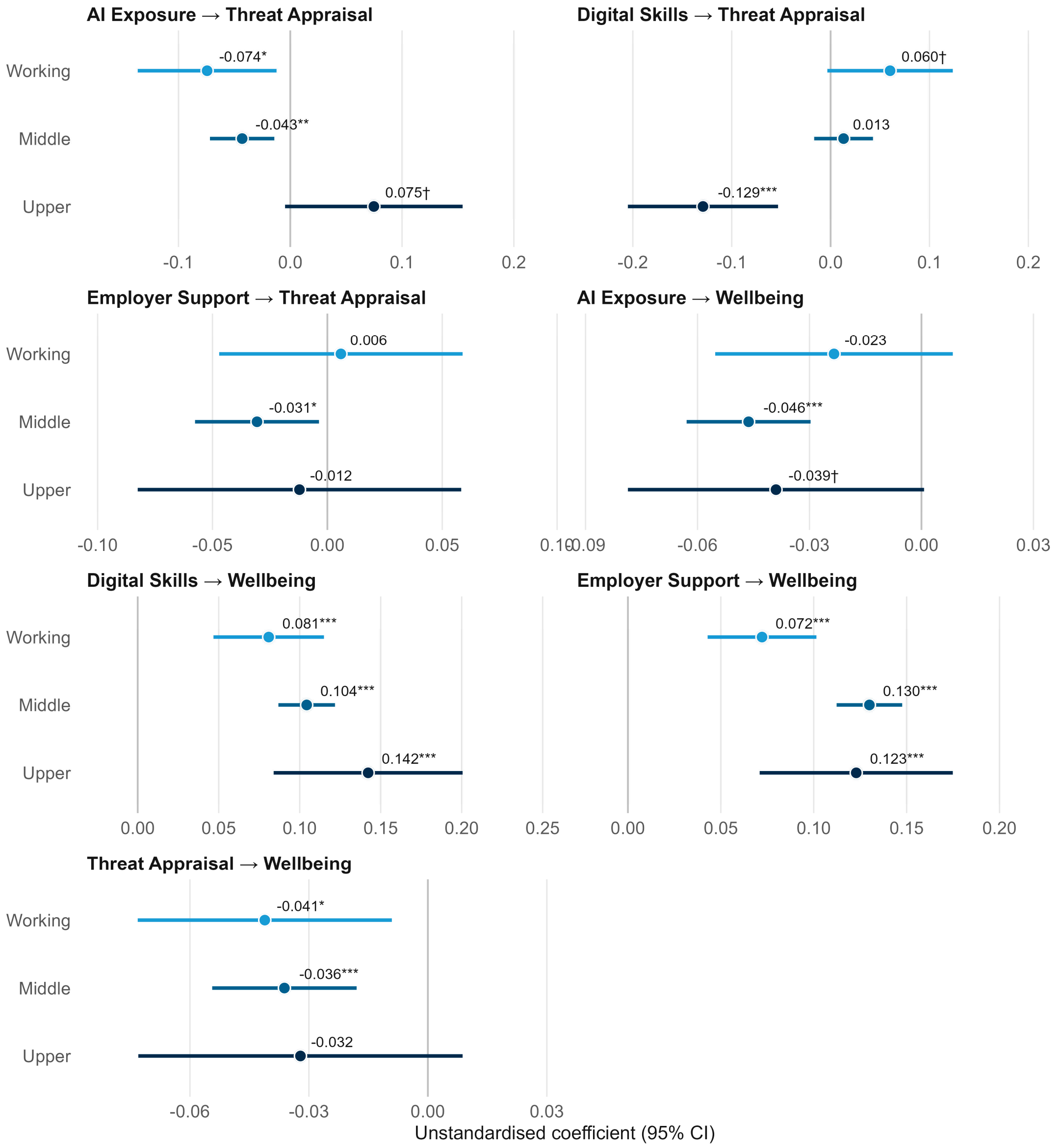

Note. N = 8,065 (working class = 1,454; middle class = 5,788; upper class = 823). Unstandardised coefficients with 95% confidence intervals from the multi-group model in which all structural paths were freely estimated. Horizontal scales differ across panels. Omnibus test that all seven paths are equal across classes: $\Delta\chi^2(14) = 33.36$, $p = .003$. Employer digital training → wellbeing differs across classes, $\chi^2(2) = 11.08$, $p = .004$; digital skills → wellbeing does not, $\chi^2(2) = 3.37$, $p = .186$. Upper-class estimates should be interpreted with caution: the solution for that group contains a Heywood case (negative residual variance on one wellbeing indicator) arising from sparse response categories. *** $p < .001$, ** $p < .01$, * $p < .05$, † $p < .10$.

Figure S3.

Robustness check: main model versus reduced model without employer digital training

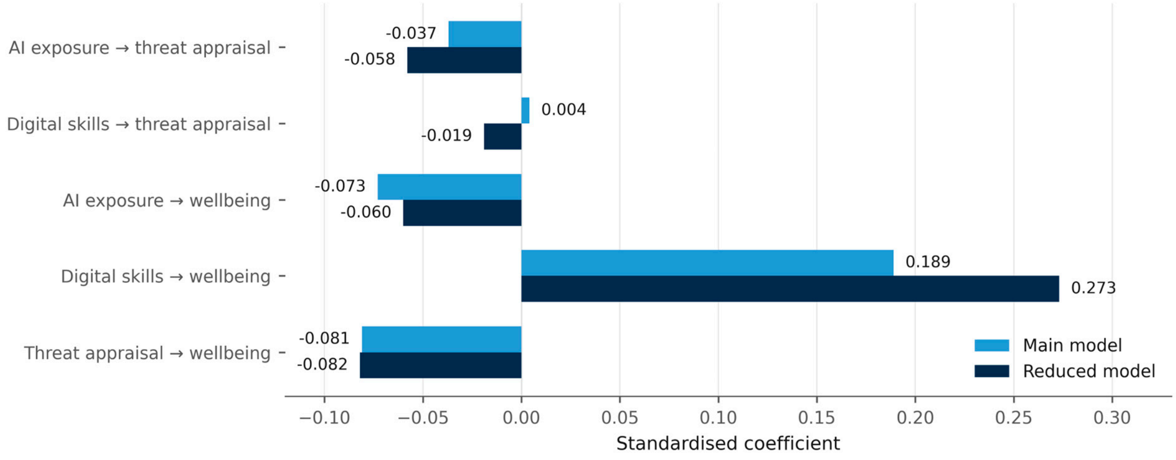

Note. Standardised coefficients from the pooled structural model (WLSMV, ordinal indicators, seven control variables). The main model (n = 8,065) includes employer digital training; the reduced model omits it, which removes the requirement of a valid answer to that item and allows the self-employed to enter (n = 9,539; Table S7). Every path retains its sign and significance across the two specifications. Model fit improves in the reduced model (CFI .967 → .988; RMSEA .082 → .056), as expected given the simpler structure.

Figure S4.

Random forest variable importance

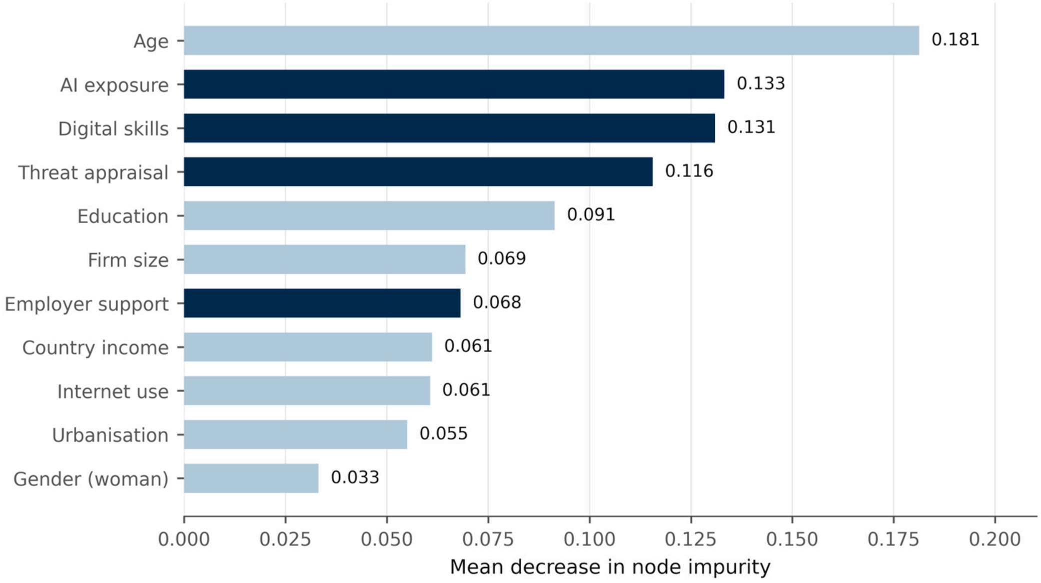

Note. n = 8,065; 500 trees; cross-validated R^2 = .159. Importance is the mean decrease in node impurity. Dark bars mark the four theoretical constructs; all four rank in the top seven of eleven predictors. This metric is not a partial effect and systematically under-weights single-indicator predictors relative to multi-item latent constructs, which is why employer digital training ranks lower here than its structural coefficient would suggest. On mean |SHAP| (Table S9), the comparable metric, employer digital training ranks second among the four theoretical constructs and third of all eleven predictors, behind digital skills and country income.

Figure S5.

Random forest variable importance by social class

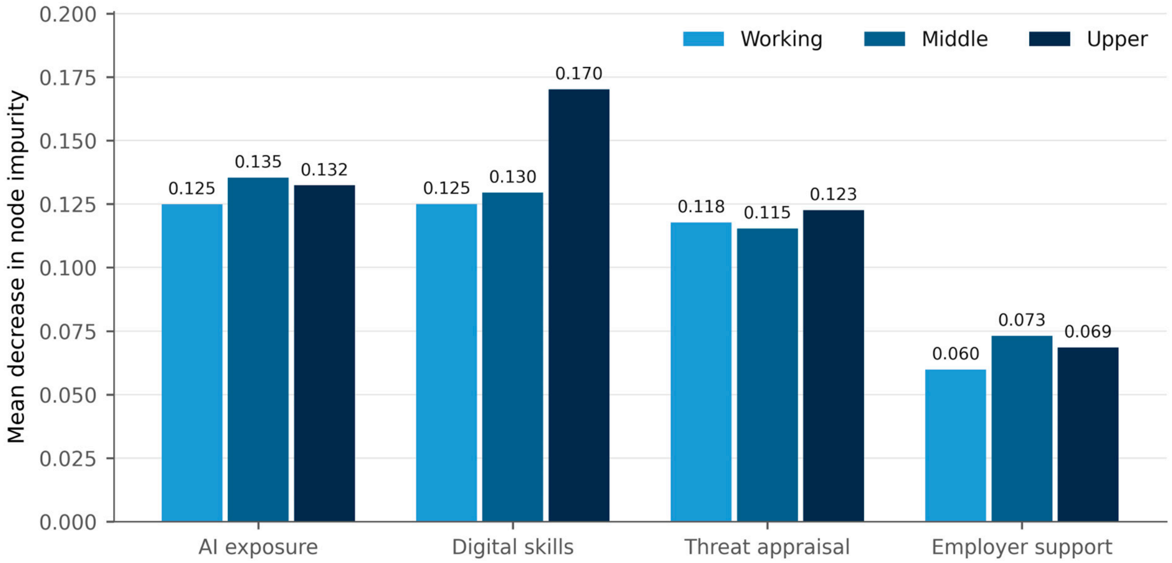

Note. Working class n = 1,454; middle class n = 5,788; upper class n = 823. Separate random forests were fitted within each class. Cross-validated R^2 was -0.007 for the working class, .152 for the middle class and .174 for the upper class; the negative value for the working class indicates out-of-sample prediction worse than the sample mean, consistent with the structural model accounting for less of working-class wellbeing. Digital skills importance rises with class position, independently reproducing the gradient reported in Table 4 and Figure S2.

Figure S6.

SHAP dependence plots for the four theoretical constructs

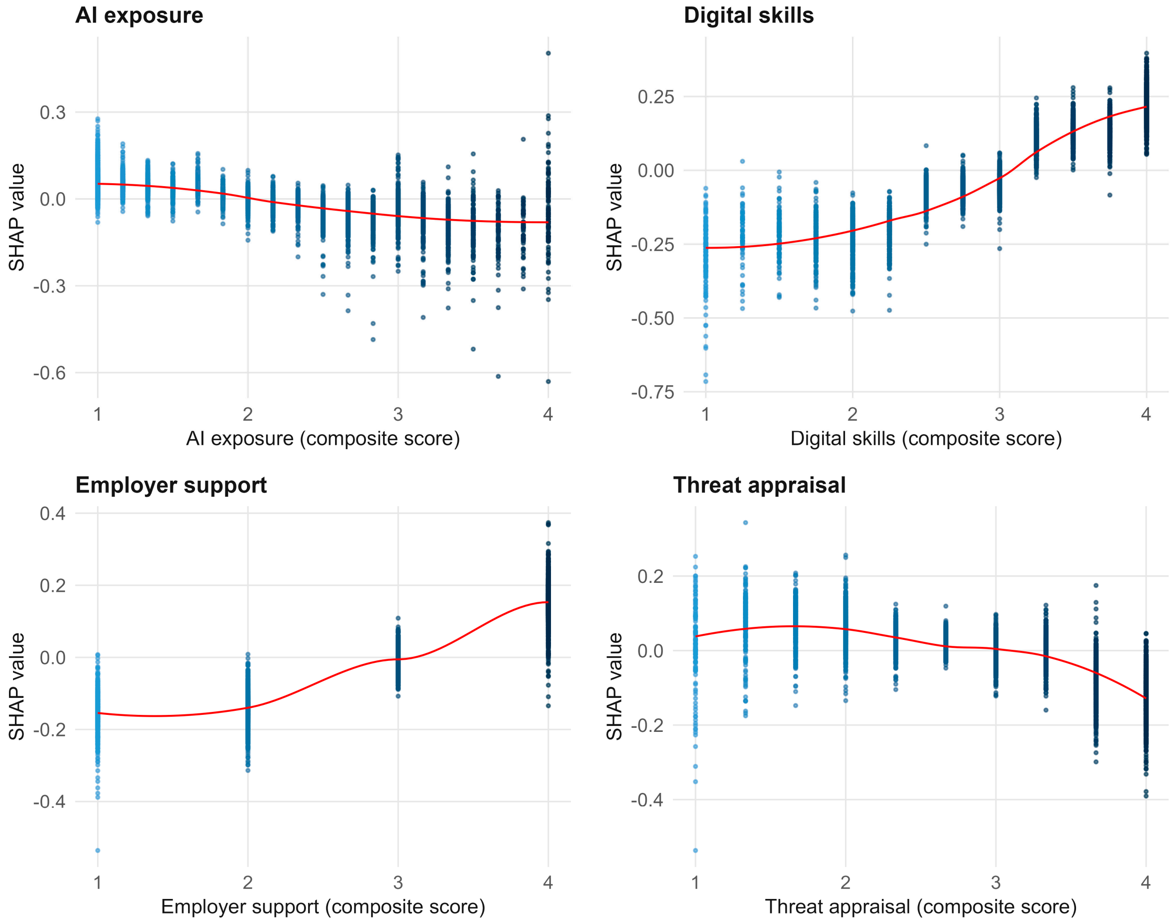

Note. n = 8,065. SHAP values from the XGBoost model reported in Figure 3 (100 rounds, maximum depth 6, learning rate 0.1). Each point is one respondent; the horizontal axis is the respondent's score on the construct and the vertical axis is that score's contribution to predicted wellbeing; the line is a smoothed trend. Employer digital training is a single four-point item, so its horizontal axis shows the item score. Digital skills and employer digital training raise predicted wellbeing across their range, threat appraisal lowers it mainly at the top of the scale, and AI exposure has a weak negative profile — the same signs as the structural model (Table 3). Mean |SHAP|: digital skills 0.133, employer digital training 0.084, threat appraisal 0.049, AI exposure 0.047.
